# Simulated millennial-scale climate variability driven by a convection–advection oscillator

**DOI:** 10.1007/s00382-025-07630-x

**Published:** 2025-03-07

**Authors:** Yvan M. Romé, Ruza F. Ivanovic, Lauren J. Gregoire, Didier Swingedouw, Sam Sherriff-Tadano, Reyk Börner

**Affiliations:** 1 School of Earth and Environment, University of Leeds, Woodhouse Lane, Leeds, LS2 9JT UK; 2 CNRS, Bordeaux INP, EPOC, UMR 5805, University of Bordeaux, Pessac, 33600 France; 3 Faculty of Science, University of the Ryukyus, 1 Senbaru, Nishihara, Nakagami District, Okinawa, 903-0129 Japan; 4 Department of Mathematics and Statistics, University of Reading, Whiteknights House, Reading, RG6 6UR UK

**Keywords:** Millennial-scale variability, AMOC, General circulation model, Salt oscillator, North Atlantic stratification, Convection–advection oscillator

## Abstract

The last glacial period, between around 115 and 12 thousand years before present, exhibited strong millennial-scale climate variability. This includes abrupt transitions between cold and warm climates, known as Dansgaard–Oeschger (D–O) cycles. D–O cycles have been linked to switches in dynamical regimes of the Atlantic Overturning Meridional Circulation (AMOC), but the exact mechanisms behind abrupt climate changes and AMOC regime shifts remain poorly understood. This paper introduces the convection–advection oscillator mechanism to explain the millennial-scale oscillations observed in a set of HadCM3 general circulation model simulations forced with snapshots of deglacial meltwater history. The oscillator can be separated into two components acting on different time scales. The fast convection component responds to changes in vertical stratification in the North Atlantic by activating or deactivating deep water formation sites. The slow advection component regulates the accumulation and depletion of salinity in the North Atlantic. This oscillator mechanism is triggered under specific background conditions and freshwater release patterns. The freshwater perturbation causes an instability that triggers a global salt reorganisation, modifying the North Atlantic stratification. For a given forcing pattern, the system oscillates if the salt transport can lead to an alternating reactivation and deactivation of the AMOC. Otherwise, the climate settles in a warm or cold steady state. This mechanism expands existing theories of millennial-scale variability and provides a general framework for understanding abrupt climate change in general circulation models.

**Supplementary Information:**

The online version contains supplementary material available at 10.1007/s00382-025-07630-x.

## Introduction

### Millennial-scale variability during the last glacial period

The Greenland ice core records of the last glacial period, stretching between about 115 and about 12 ka BP (thousand years before present), are dominated by millennial-scale variability (Wolff et al. 2010). Dansgaard–Oeschger (D–O) cycles (Dansgaard et al. 1993; Bond et al. 1993), recurring transitions between cold *stadial* and warm *interstadial* North Atlantic climates (Thomas et al. 2009; Lohmann and Ditlevsen 2019), are the most prominent example of such variability. During these transitions, Greenland underwent temperature changes of up to ${16\,\mathrm{^{\circ }C}}$ (Huber et al. 2006; Kindler et al. 2014; Buizert et al. 2014) within a few decades (Rasmussen et al. 2014). North Atlantic abrupt climate changes have been widely associated with changes in the Atlantic Meridional Overturning Circulation (AMOC, Rahmstorf 2002; Clark et al. 2002). This is primarily because the AMOC is believed to have shifted between different modes during the last glacial period (Broecker et al. 1985; Gottschalk et al. 2015; Henry et al. 2016; Lynch-Stieglitz 2017; Weijer et al. 2019), disrupting the flux of heat and salt into the North Atlantic. Three dynamical regimes of the AMOC have been hypothesised by Rahmstorf (2002): a *warm* mode where a vigorous AMOC reaches up to 70$^{\circ }$N, a *cold* mode where a shallower AMOC does not extend past the Greenland-Iceland-Scotland (GIS) ridge, and an *off* mode where the AMOC is collapsed (Böhm et al. 2015). Simulations of the climate of the next centuries (Weijer et al. 2020; Romanou et al. 2023) and observed fingerprints of potential decline in AMOC strength (Thornalley et al. 2018; Caesar et al. 2018; Boers 2021; Michel et al. 2022; Ditlevsen and Ditlevsen 2023) have raised concerns that the AMOC might undergo an abrupt weakening or collapse again with potentially dramatic consequences on the North Atlantic climate (van Westen et al. 2024).

However, the climate projections are highly model-dependent (Weijer et al. 2020; Bellomo et al. 2021) and direct observations are still too short to draw any statistically significant conclusion on potential shifts in the AMOC regime (Lobelle et al. 2020; Jackson et al. 2022). Reducing the uncertainty in future AMOC stability requires a deeper understanding of the dynamic processes driving the overturning circulation, which can be examined through climate modelling of the last glacial period. In particular, the Last Glacial Maximum (LGM)—about 21 ka BP—and marine isotope stage 3—about 60–30 ka BP—have proven insightful for studying AMOC stability (Weijer et al. 2019). Freshwater hosing experiments have historically been an effective way to trigger abrupt climate changes in most climate models (Manabe and Stouffer 1995; Kageyama et al. 2010; Hawkins et al. 2011; Kageyama et al. 2013; van Westen and Dijkstra 2023). When it comes to simulating spontaneous oscillations, however, General Circulation Models (GCMs) were for a long time regarded as too biased towards producing more stable climates (Valdes 2011; Mecking et al. 2017; Liu et al. 2017). This bias was addressed by making use of modern supercomputers to explore a comprehensive range of model inputs (including uncertain model parameters and climate forcings) and running models for multiple millennia. As a result, more GCMs have been found to exhibit millennial-scale variability in the past decade (e.g., Peltier and Vettoretti 2014; Brown and Galbraith 2016; Klockmann et al. 2018; Zhang et al. 2021; Armstrong et al. 2022; Kuniyoshi et al. 2022; Malmierca-Vallet et al. 2023).

Barker and Knorr (2021) coined the term ‘window of opportunity’ to define a model parameter regime where millennial-scale variability can occur. Not all models share the same windows of opportunity, i.e. the location and extent of the window of opportunity in the parameter space varies across models. Most models agree, however, on the most relevant parameters that determine its definition; namely the atmospheric greenhouse gases, orbital parameters, ice sheet topography and freshwater release. Atmospheric CO$_2$ concentrations (Brown and Galbraith 2016; Zhang et al. 2017; Klockmann et al. 2018; Vettoretti et al. 2022; Malmierca-Vallet et al. 2024) and insolation (Zhang et al. 2021; Kuniyoshi et al. 2022) are essential to the creation of conditions facilitating abrupt climate change through their influence on the Atlantic energy balance, atmospheric and oceanic circulations and stratification. The ice sheet configuration is equally important, as the extensive ice sheets covering North America and northern Europe during the last glacial maximum (Hughes et al. 2016; Batchelor et al. 2019) act to prevent the occurrence of AMOC mode shifts (Zhang et al. 2014; Brown and Galbraith 2016; Klockmann et al. 2018). Glacial ice sheets tend to drive a less variable, more southern and potentially faster eddy-driven jet (Merz et al. 2015; Li and Born 2019). This results in stronger surface winds and wind stress curl that intensify the salt and heat transport to the deep water formation sites (Czaja 2009; Montoya et al. 2011; Sherriff-Tadano et al. 2018, 2021). The topography and albedo of the ice sheets also modify the inter-hemispheric heat fluxes (Roberts and Valdes 2017). In addition, glacial ice sheets can produce larger freshwater fluxes than the pre-industrial ice sheers, as observed in sea level records of the last glacial period (Fairbanks 1989; Lambeck et al. 2014). This is another critical input because freshwater can enhance ocean stratification by decreasing the surface layer density, reducing deep convection and thereby weakening the AMOC. Freshwater forcing is particularly efficient at slowing down the AMOC when targeted at regions of deep water formation, such as within the Nordic Seas (Smith and Gregory 2009; Roche et al. 2010), but the exact rate and location of freshwater release during the last glacial period discharge events remains challenging to constrain (Birchfield et al. 1994; Bethke et al. 2012).

### Mechanisms of millennial-scale variability

With his pioneering box model, Stommel (1961) demonstrated that heat and salt fluxes between two interconnected basins could lead to two distinct stable regimes of ocean circulation. This hypothesis was influential in the establishment of theories for AMOC regime shifts in the context of millennial-scale variability. As an alternative to multistability, Broecker et al. (1990) introduced the salt oscillator as a mechanism for millennial-scale variability. In this theory, when the AMOC is weak, salt is accumulated in the Atlantic by evaporation and increases the surface density, leading to the reactivation of North Atlantic vertical convection and the overturning circulation. When the AMOC is strong, the overturning circulation exports salt out of the Atlantic basin and the surface density decreases, leading to the deactivation of North Atlantic deep water formation sites and a weakening of the overturning circulation. The validity of this mechanism depends on the sign of the net salt export out of the Atlantic Ocean (Huisman et al. 2010; Drijfhout et al. 2011), and an initial perturbation is required to start the oscillator. Millennial-scale variability can also arise from local instabilities. Spontaneous oscillations in a simple mixed-layer model were demonstrated by Welander (1982), and Cessi (1996) argued that a freshwater input could trigger this oscillator. Winton and Sarachik (1993) also explored the role of vertical instability of the water column to introduce the concept of deep-decoupling oscillations. Deep-decoupling oscillations rely on the different response times between the surface and the deep North Atlantic waters, and the building and collapse of the halocline in the North Atlantic deactivates or reactivates deep convection. Colin de Verdière (2007) proposed a model where the salt-oscillator theory described by Broecker et al. (1990) and the vertical mixed-layer oscillations of Welander (1982) work in concert to produce millennial-scale oscillations. Finally, stochastic resonance was suggested as another possible mechanism to trigger abrupt regime shifts (Alley et al. 2001; Ganopolski and Rahmstorf 2002; Cimatoribus et al. 2013). This theory relies on stochastic noise to amplify internal ocean variability and kick-start oscillations (Schulz et al. 2002; Timmermann et al. 2003).

An effective framework for comparing complex climate model simulations with theoretical models is to conceptualise millennial-scale variability mechanisms as a combination of slow and fast components (Roberts and Saha 2017; Vettoretti et al. 2022). Slow components modify the atmospheric and oceanic conditions in the North Atlantic, while fast components consist of a combination of thresholds and positive feedbacks that can rapidly alter deep water formation activity. Although the scope and definition of the salt oscillator has varied since its introduction (Broecker et al. 1990), it remains a central concept to describe the slow components (e.g. Vettoretti and Peltier 2018; Klockmann et al. 2020; Armstrong et al. 2022). Vettoretti and Peltier (2018) showed that southwards transport of Arctic sea ice was responsible for the freshening of the North Atlantic prior to a cooling event, and the northwards gyre transportation of salt into the North Atlantic was responsible for the salinification of the North Atlantic prior to a warming event. In Klockmann et al. (2020)’s simulations, phases of accumulation and depletion of the subtropical Atlantic salinity affect the density gradient across the subpolar gyre. Similar to Broecker et al. (1990), Armstrong et al. (2022)’s analysis points towards changes in Atlantic precipitation and evaporation patterns due to shifts of the ITCZ. Additionally, they discuss the influence of sea ice transport on the meridional salinity gradients in the North Atlantic. In Kuniyoshi et al. (2022)’s study, sea ice transport is also the main driver of the mechanisms, although thermal changes of the North Atlantic water column are more impactful than changes in surface salinity gradient. Thermal control of millennial-scale oscillations was equally considered in previous works (e.g., Oka et al. 2012; Dokken et al. 2013; Brown and Galbraith 2016). Additionally, sources of salt and heat outside of the North Atlantic, such as the Southern Ocean (Knorr and Lohmann 2003; Banderas et al. 2015; Buizert and Schmittner 2015; Thompson et al. 2019; Oka et al. 2021) or the Indian Ocean (Nuber et al. 2023), were also identified as potential drivers of slow changes of the North Atlantic water masses. This is the case, for instance, in the mechanism by Vettoretti et al. (2022) where the density of the AABW modifies the Atlantic vertical stratification. Finally, Weijer and Dijkstra (2003) argues that a salinity anomaly that propagates along the different ocean basins could set the pacing of the oscillations.

The fast dynamics involve positive feedback that can accelerate the reactivation and deactivation of the deep water formation sites when one or several North Atlantic climate thresholds. The salt-advection feedback (Drijfhout et al. 2015; Weijer et al. 2019) is a central concept here: a stronger AMOC advects more salt to the deep water formation sites, enhancing deep convection which further amplifies the AMOC. Conversely, a decrease in AMOC strength further weakens the AMOC by advecting less salt northwards, reducing surface density at the deep convection sites. This is at stake, for instance, in Kuniyoshi et al. (2022) and Vettoretti and Peltier (2018)’s mechanism, where the positive feedback accelerates the initial changes in North Atlantic deep water formation when the vertical stratification in the region passes a certain threshold. In other instances, the feedback can amplify the impact of exceptional events, such as the opening of polynyas to release abruptly the heat accumulated under the surface of the ice (a phenomenon referred to as subsurface warming) in Vettoretti and Peltier (2016). Both the threshold and exceptional events approaches were considered in Klockmann et al. (2020), building on the theory introduced by Li and Born (2019), to describe a positive feedback between the winds and the subpolar gyre leading to shifts in the North Atlantic convection. This feedback can be activated either by a change in the cross-gyre density gradient or by stochastic wind forcing. In Armstrong et al. (2022), the meridional density gradient in the North Atlantic acts as the threshold, and both the wind-driven and the salt advection feedbacks accelerate the changes in North Atlantic convection. Kuniyoshi et al. (2022) describes a sea-ice positive feedback that cools down the North Atlantic during an interstadial to stadial transition. Finally, external forcing such as the freshwater input used in Brown and Galbraith (2016) and the change of vertical diffusivity in Peltier and Vettoretti (2014) can act as the initial trigger of the fast components.

### Objectives of the paper

The Hadley Centre Climate Model 3, also known as HadCM3 (Valdes et al. 2017), is one of the models that has managed to simulate abrupt climate changes under realistic glacial conditions. Signs of AMOC transitions were previously reported in response to freshwater forcing (Matero et al. 2017; Ivanovic et al. 2018), but only recently did two independent studies manage to observe oscillations of the North Atlantic climate: Romé et al. (2022) triggered oscillations using deglacial meltwater patterns in a last glacial maximum background state and Armstrong et al. (2022) described oscillations that occur for a specific snapshot of marine isotope stage 3 conditions (30 ka BP boundary conditions). In this work, we aim to analyse the processes at stake in the LGM simulations presented in Romé et al. (2022) and explore how they connect to other mechanisms derived from HadCM3 and other model simulations. We do not try to directly identify the mechanisms behind D–O cycles, as the experimental design prevents a direct comparison. After presenting the experimental design of the simulations (Sect. 2) and summarising the results and terminology introduced by Romé et al. (2022) (Sect. 3), we separately examine the changes in stratification at the North Atlantic convection sites (Sect. 4.1) as well as the global salt exchanges (Sect. 4.2). Section 5 brings these insights together to introduce the convection–advection oscillator mechanism. Section 6 analyses how our mechanism applies to non-oscillating simulations and could help characterise AMOC stability in general circulation models. Finally, Sect. 7 explores the limitations of this theory and how it connects to previous mechanisms for millennial-scale variability.

## Methods

### The HadCM3 general circulation model

All the simulations in this study used the BRIDGE (Bristol Research Initiative for the Dynamic Global Environment group) version of the HadCM3 (HadCM3B) atmosphere-ocean general circulation model developed by Valdes et al. (2017). The atmosphere model, described by Pope et al. (2000), consists of a $2.5^{\circ }$ x $3.75^{\circ }$ grid on 19 elevation levels. The ocean model, described by Gordon et al. (2000), is a $1.25^{\circ }$ x $1.25^{\circ }$ grid on 20 depth layers. In order to verify the equation of state (Bryan and Cox 1972; Fofonoff and Millard 1983; Fofonoff 1985), the ocean model uses a rigid surface lid (Gordon et al. 2000), meaning that the ocean volume stays constant throughout the simulations. In addition to the ocean and atmosphere components, the model includes the MOSES 2.1 land model (Cox et al. 1999) and the TRIFFID dynamic vegetation model (Cox 2001). The land-sea mask is matched to the chosen palaeo ice sheet reconstruction. The Gibraltar Strait is closed during the LGM—as it is for the present, due to the spatial resolution of the model—and Mediterranean-Atlantic salt and heat exchanges are parametrised by a diffusive pipe (Ivanovic et al. 2013, 2014). HadCM3B was optimised for multi-millennial palaeoclimate runs (Valdes et al. 2017) and has been used previously for glacial simulations with an imposed meltwater forcing (e.g., Ivanovic et al. 2018; Romé et al. 2022).

### Global mean salinity conservation

HadCM3 uses a rigid-lid ocean model to compute water fluxes. The rigid-lid model implies that the volume of the ocean remains constant throughout the simulations, and water fluxes, whether they are freshwater discharge, precipitation or evaporation, are implemented in the model as a change of the surface layer salinity rather than a physical input of water. This also means that the land-sea mask is not updated in response to the freshwater fluxes in such experiments.

This method does not guarantee the closing of the hydrological budgets causing shifts in the global mean salinity. Global mean salinity drifts of the order of ${0.25\,\mathrm{g.kg^{-1}.kyear^{-1}}}$ can spontaneously occur in HadCM3 even without freshwater input (Dentith et al. 2019). In other cases, global salinity increase can also be observed if the model configuration is biased towards higher evaporation than precipitation, leading to salt accumulation in inland seas and the tropical oceans. On top of these background shifts in global salinity, an input of meltwater of around ${0.1\,\textrm{Sv}}$, the order of magnitude of the discharge used in this study, can drive a significant decrease in salinity comparable to that observed during the last deglaciation over 10.000 years of simulations.

The aim of this set of simulations is not to simulate a deglaciation and the continued freshening of the global ocean during the long integration should be avoided. To tackle this issue, we use a global salinity conservation algorithm—this corresponds to the *VFLUX* method described by Dentith et al. (2019). The VFLUX algorithm sets a global mean salinity target (shortened salinity target in the following) for the entire run. At the end of each time step, the model corrects the salinity uniformly throughout each depth so that the corrected global mean salinity matches the value of the salinity target. The global mean salinity target is calculated from the 1950 CE (Common Era) global mean salinity, adjusted to account for the differences in the ice sheets volume between the present and a given point in time. Note that the change in salinity target only covers ice volume changes and does not take into account additional freshwater input. The new salinity target is implemented at the start of each simulation, and a transient regime where the model adjusts to the new conditions can be observed in the initial millennium (Romé et al. 2022).

### Experimental design

The simulations in this paper, presented in Table 1, are branched from the *CTRL_LGM* (or *control*) simulation at year 0. The *control* simulation is a last glacial maximum run following the 21 ka BP PMIP4 protocol for the boundary conditions (greenhouse gas concentrations, orbital parameters and solar constant, Kageyama et al. 2017). We used the GLAC-1D (Tarasov and Peltier 2002; Tarasov et al. 2012; Briggs et al. 2014; Ivanovic et al. 2016) ice sheet reconstruction and the corresponding land-sea mask, orography and bathymetry. *CTRL_LGM* extends a previous HadCM3 last glacial maximum simulation (Davies-Barnard et al. 2017), and starts after an initial 3,500 years spin-up with the updated boundary conditions. *CTRL*_*LGM*’s start year will be the year 0 in our time series.

**Table 1:** Experiments summary

Simulation	Meltwater snapshot	Integration length	Salinity Target	Tendencies
name	*Meltwater flux (Sv)*	(years)	($g.kg^{-1}$)	(included)
*CTRL_LGM*	None	4000	35.8334	No
*20.7k*	20.7 ka BP *(0.084)*	10,000	35.8225	No
*21k*	21.0 ka BP *(0.054)*	4000	35.8334	No
*18.2k*	18.2 ka BP *(0.109)*	10,000	35.7348	No
*20.7k_tdc*	20.7 ka BP *(0.084)*	4000	35.8225	Yes
*20.7k_no_gst*	20.7 ka BP *(0.084)*	3000	None	No

All experiments were designed with LGM boundary conditions, using the LGM GLAC-1D ice sheet extent and associated geographies. The *meltwater* entries indicate the meltwater snapshot used (see Sect. 2.3) and the total meltwater flux in brackets. The meltwater flux only corresponds to the freshwater from the ice sheet melting and does not include the other freshwater fluxes (e.g., precipitation, sea ice, etc.). The *Salinity Target* (g.kg$^{-1}$) entries are the global salinity target used for the salinity correction described in Sect. 2.2. *Tendencies* indicates if the salinity tendencies diagnostic described in S6 were included

The main focus of this paper will be the *20.7k* simulation described in Romé et al. (2022). The *20.7k* simulation differs from *CTRL_LGM* only by the addition of a **constant** meltwater discharge corresponding to the **20.7 ka BP** slice of GLAC-1D meltwater history derived by the protocol introduced in Romé et al. (2022). For each time step of the GLAC-1D ice sheet reconstruction, freshwater discharge was computed from changes in ice sheet elevation at each grid cell and routed to an ocean grid cell using an offline drainage map of the last glacial maximum (Ivanovic et al. 2016). Surface freshwater saturation (i.e. a grid cell salinity of ${0\,\textrm{g}.\mathrm{kg^{-1}}}$) can be reached in some grid cells when large freshwater fluxes are recorded. To avoid this situation, the meltwater is spread across a wider (proximal ocean) region around the source grid box. Spreading regions are in the order of ${10\,\mathrm{^6km^{2}}}$, about a hundred ocean grid cells, an area comparable to a typical Heinrich event (Roberts et al. 2014). From this meltwater forcing history, we derive the freshwater pattern corresponding to the time slice we are interested in, in this case 20.7 ka BP. This pattern will be called the meltwater snapshot and used as a constant forcing for the 10,000 integration years. Independently from the freshwater discharge, we change the global mean salinity target to match the meltwater snapshot, in this case ${35.8255\,\textrm{g}.\mathrm{kg^{-1}}}$ for 20.7 ka BP.

To get a more precise picture of the ocean dynamics in our simulations, we repeated the *20.7k* simulation for 4000 years, saving an extra set of salinity diagnostics, or salinity tendencies, in the model output. This simulation is called *20.7k_tdc*. Armstrong et al. (2022) introduced the HadCM3 tendencies diagnosis and included a comprehensive description of the implementation. We summarise the essential information in Section S6.

We included two additional simulations from Romé et al. (2022) for comparison. The *21k* simulation used the meltwater snapshot corresponding to 21 ka BP, a global salinity target of ${35.8334\,\textrm{g}.\mathrm{kg^{-1}}}$ and was run for 4000 years. The *18.2k* simulation used the meltwater snapshot corresponding to 18.2 ka BP, a global salinity target of ${35.7348\,\textrm{g}.\mathrm{kg^{-1}}}$ and was run for 10,000 years. Finally, we investigated the impact of the global salinity target by running a simulation with the salinity correction algorithm turned off. This simulation, called *20.7k_no_gst*, used the same meltwater snapshot as *20.7k* and was run for 3000 years.

## Anatomy of the simulations

**Fig. 1:**
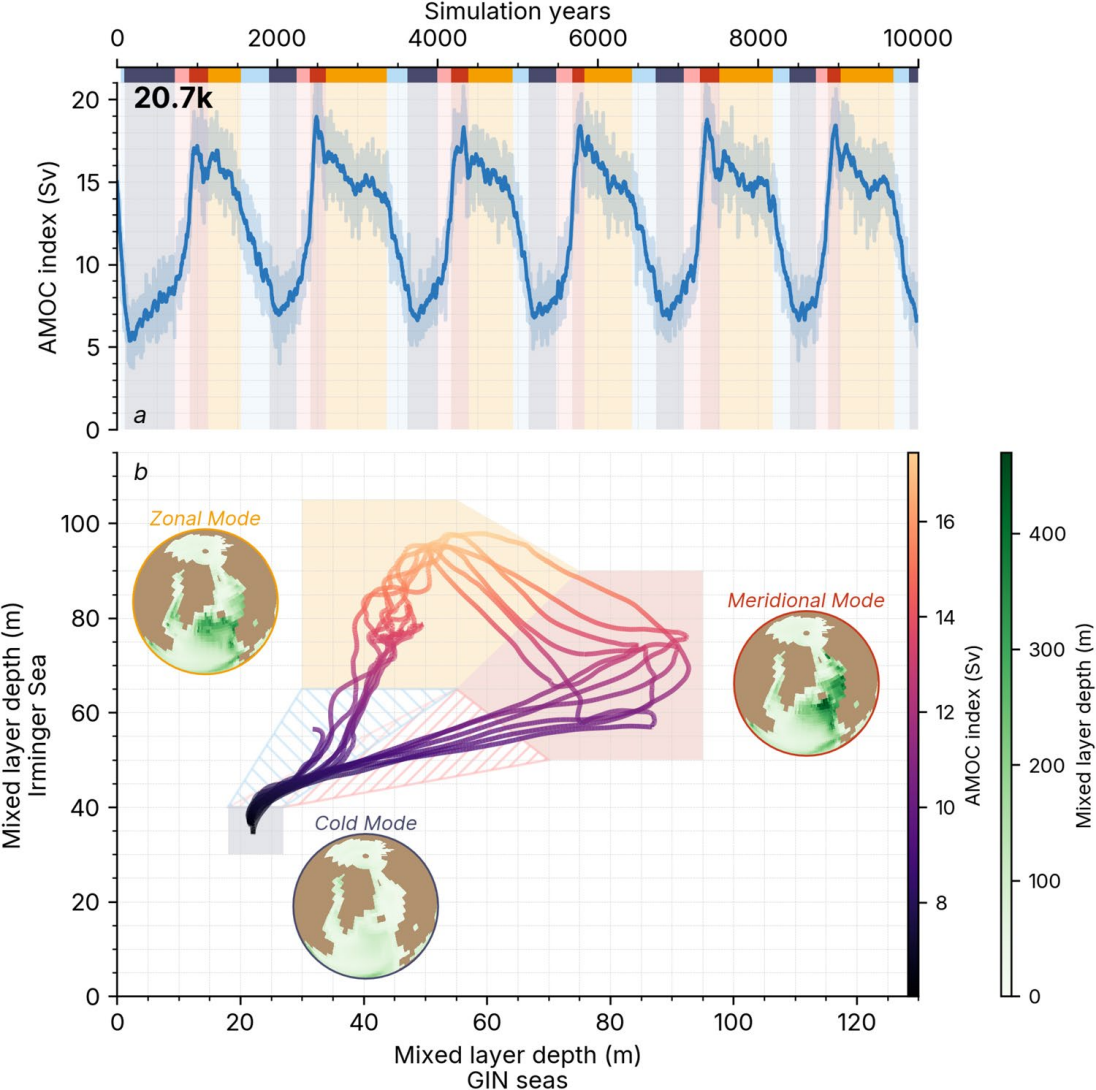
Anatomy of the oscillations. **a** Time-series of AMOC index (maximum overturning circulation at $26.5^{\circ }\,\hbox {N}$) in the *20.7k* simulation. The background colour bars indicate the phases with *cold* in dark blue, *warming* in pink, the *meridional* in red, *zonal* in orange and *cooling* in light blue. Solid lines indicate the 30-year running mean and transparent lines the annual mean. **b** State space visualisation (i.e. scatter-plot) of the annual mean mixed layer depth in the Greenland Iceland Nordic (GIN) Seas against the mixed layer depth in the Irminger Sea (the zones are defined in Figure S1b). Each point corresponds to a year of the *20.7k* simulation time series after being processed by a low-pass filter with a cut-off frequency of ${2\,10^{-3}year^{-1}}$—the algorithm was described in Section S3. Shaded boxes define the phases shown in panel *a*. In the small globes, the composite mean winter (DJF) mixed layer depth is plotted for the *cold* and the two *warm* phases

Romé et al. (2022) introduced a method for clustering the meltwater snapshot simulations between different convection modes characterising the North Atlantic deep water formation layouts. An *oscillating* simulation is characterised by the periodic alternation of ‘warm’ modes—vigorous AMOC with relatively warm North Atlantic temperatures—and ‘cold’ modes—weak AMOC with relatively cold North Atlantic temperatures. The *20.7k* simulation is an example of an *oscillating* simulation where this sequence can be observed in Fig. 1a. If instead of sustained quasi-oscillations the AMOC settles in a warm or cold steady state, the simulation is referred to as a *warm* or *cold* simulation, respectively. The aim of Romé et al. (2022) was not to reproduce D–O cycles and we will therefore avoid using the commonly applied ‘stadial’/‘interstadial’ terminology. Nonetheless, it can be helpful to think of warm and cold modes in the light of interstadials and stadial states.

To characterise a simulation, we can look at its path in the state space projected onto the Mixed Layer Depth (MLD)—an indicator of the mixing and convection of the upper water column—in the GIN Seas against the MLD in the Irminger Sea (as plotted in Fig. 1b). A detail of the zones is given in Figure S1*b*. In this state space projection (Fig. 1b), we manually define five distinct *phases* of the oscillatory cycle. The *cold* phase, where deep convection is weak in the North Atlantic; the *meridional warm* phase (or simply *meridional* phase) with a main convection region in the GIN Seas and an intermediate convection spot in the Irminger Sea; the *zonal warm* phase (or simply *zonal* phase) dominated by convection in the Irminger Sea and low convection in the GIN Seas; the *warming* phase, a transition from the *cold* phase to the *meridional* phase and the *cooling* phase, a transition from the *zonal* phase to the *cold* phase. In this paper’s simulations, the transition from the *meridional* to the *zonal* phase is too fast to be considered a distinct phase. Each phase was assigned a colour, and coloured bars will be shown in time series plots of this article in the style of Fig. 1a.

The *oscillating 20.7k* simulation evolves along a well-defined cycle with a periodicity of about 1540 years (see Section S3). After an initial drop to a *cold* phase, it starts by *warming* from the *cold* phase to the *meridional* phase, switching to the *zonal* phase after a few hundred years before *cooling* back to the *cold* phase. The surface temperature in Greenland typically varies by ${8\,\mathrm{^{\circ }C}}$ over the course of an oscillation (Romé et al. 2022). It takes a couple of cycles for the pattern to become more regular as the climate system has completely adjusted to the freshwater perturbation and the change of salinity target, and we will therefore refer to the first $\sim$ 4000 years as the *transient* regime.

The AMOC time series and state space plots of the *18.2k*, *21k*, *20.7k_no_gst* and *20.7k_tdc* simulations are shown in Figure S2. Across all simulations, the modes are associated with the same convective dynamics as the phases of the oscillators, although they span slightly different areas on the two-dimensional MLD state space projection due to the different freshwater release patterns. Note that in the general case, we call a *warming* mode a transition between a *cold* mode to any *warm* mode and a *cooling* mode from any *warm* mode to a *cold* mode. The *CTRL_LGM* simulation is a *warm* simulation at a steady state in a *zonal* mode. All meltwater release simulations respond to the initial meltwater release with a multi-centennial drop of the AMOC. The *21k* simulation then recovers into a *zonal* steady state and thus is classified into the *warm* simulations, similarly to the *20.7k_no_gst* simulation that follows one oscillatory cycle before stabilising in a *zonal* steady state. On the other hand, the *18.2k* simulation mostly remains in a *cold* steady state after the initial perturbation, albeit interrupted by short-lived excitations into the *meridional* mode. Because of the irregular and transient nature of these excitations, this simulation is categorised as a *cold* simulation. Finally, even though the *oscillating* *20.7k* and *20.7k_tdc* simulations are effectively the same experiment, the noise introduced when the model was restarted to initialise the salinity tendencies diagnostics (a process called ‘reconfiguration’) results in slight differences in the periodicity and shape of the oscillations (Figure S2a, e, f, j). This is due to the chaotic nature of the climate system, and we consider the two simulations dynamically equivalent.

## Results

### Stratification changes at the North Atlantic deep water formation sites

**Fig. 2:**
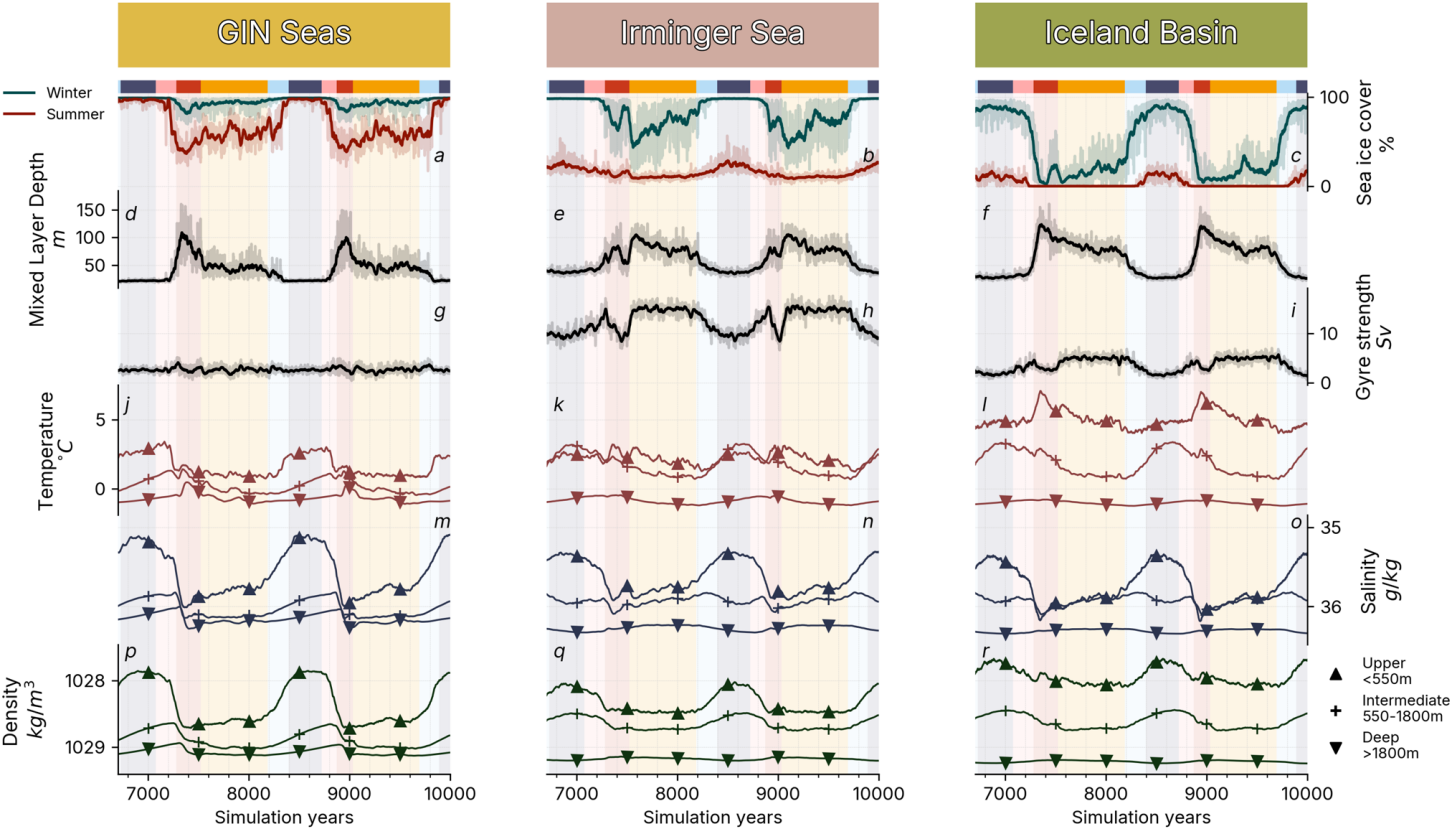
North Atlantic dynamics at deep water formation sites. Plotted for the *20.7k* simulation in the GIN Seas (left), the Irminger Sea (middle) and the Iceland Basin (right). The geographical zones are defined in Figure S1, and the background shading corresponds to the phases defined in Fig. 1. **a**–**c** Early spring (March) and early autumn (September) sea ice concentration. **d**–**f** Annual mean mixed layer depth. **g**–**i** Annual mean anti-clockwise barotropic stream function indicating gyre circulation strength. **j**–**l** Annual mean temperature in the upper, intermediate and deep depth slices. **m**–**o** Annual mean salinity in the upper, intermediate and deep depth slices. **p**–**r** Annual mean density in the upper, intermediate and deep depth slices. In panels **a**–**i**, solid lines indicate the 30-year running mean and transparent lines the annual mean

The strength of the AMOC depends on the intensity of the convection at the North Atlantic deep water formation sites, which in turn depends on the vertical stratification of the water column. The stratification can be inferred from the density profile in Fig. 2p-r, plotted here for the *20.7k* simulation between the *upper* waters (above 550 ms deep) the *intermediate* waters (550 to 1800 meters deep), and the *deep* waters (below 1800 meters deep). The depth slices were chosen so that the *intermediate* waters always lie outside of the mixed layer boundaries while still feeling the influence of the upper waters’ changes in temperature and salinity. In this section, we focused our analysis on the GIN, Irminger and Iceland basins (Figure S1b). We only included the last two cycles of the simulation to ensure minimum interference with the transient regime at the start of the simulations. A comprehensive mapping of the North Atlantic and Arctic ocean profiles is included in Figure S4. We consider the conclusions of the following analysis to be the same between the Arctic basin and the Nordic Seas, and the Labrador Sea and the Irminger Sea.

During the ***cold*** phase, **high stratification** prevents deep convection in the North Atlantic. The deep water formation (diagnosed through mixed layer depth, Fig. 2d–f) and the North Atlantic subpolar gyre strength (Fig. 2g, h) are at their weakest. Winter sea ice extends down to the Iceland Basin, and the Nordic seas are covered with ice all year long (Fig. 2a–c). The low density of the upper North Atlantic (Fig. 2p–r) is due to a deficit in salinity (Fig. 2m, n), as well as higher temperatures in the GIN and Irminger seas (Fig. 2j, k). This high stratification tends to decrease throughout the *cold* phase, and in particular in the GIN Seas through the combined effect of subsurface warming (warming under the mixed layer, most evident in intermediate waters temperatures) over the entire water column (Fig. 2j), a salinity increase of the upper waters and a salinity decrease of the intermediate and deep waters (Fig. 2m).

During the ***warming*** phase, the **stratification decreases** abruptly. The salinification of the upper GIN Seas gains momentum while the intermediate and deep waters keep freshening (Fig. 2m). As deep convection recovers (Fig. 2d) and sea ice retreats (Fig. 2a), the heat accumulated under the ice is evacuated to the atmosphere (Fig. 2j) further accelerating the stratification decrease (Fig. 2p). Similar effects are observed in the Irminger Sea (Fig. 2q) and in the Iceland Basin (Fig. 2r), although the changes in the density profile are less abrupt.

At the end of the warming phase, deep water formation resumes in the North Atlantic (Fig. 2d–f), marking the start of the ***warm*** phases characterised by **low stratification**. At the onset of the ***meridional*** phase, the subpolar gyre is reactivated (Fig. 2e) and sea ice cover reaches a minimum in the GIN Seas and the Iceland Basin (Fig. 2a, c). The density gradient reduces between the upper and deep waters (Fig. 2p–r), mostly driven by a drop in the upper waters salinity (Fig. 2m–o), salinity gradient, resulting in a minimum of stratification in the entire North Atlantic. In the GIN Seas, this minimum only lasts for a few hundred years before upper salinity starts to decrease again (Fig. 2m, p). This decline eventually leads the deep convection to shut down (Fig. 2d), preceding a sharp recovery of the subpolar gyre strength and of the Irminger Sea deep water formation, marking the start of the ***zonal*** phase. During the *zonal* phase, the slow decrease of upper waters salinity is compensated for by a reduction of the temperatures (Fig. 2j–o), and the density profiles are mostly unaltered (Fig. 2q–s). Sea ice extent, however, is consistently increasing both in summer (Fig. 2a) and winter (Fig. 4.1b, c). Disruptions appear at the end of the *zonal* phase, when the salinity decrease in upper waters intensifies (Fig. 2o) and is no longer compensated for by subsurface heat evacuation (Fig. 2l).

This triggers the start of the ***cooling*** phase where **stratification increases** abruptly. The subpolar gyre shrinks (Fig. 2e), subsurface heat accumulation restarts (Fig. 2d–f) and the salinity decreases further in the upper waters (Fig. 2m–o). The intermediate waters density reduction does not match the pace of the upper waters (Fig. 2p–r), leading to a collapse of deep convection in the North Atlantic (Fig. 2d–f) and a return to a ***cold*** phase.

In this section, we showed that stratification changes are due to the combined effect of salinity fluxes and subsurface heat accumulation at the deep water formation sites. While the thermal effect can be linked to changes in sea ice cover, there is no obvious explanation for the vertical salinity changes through dynamical processes within the North Atlantic. A deeper analysis of the global salinity transfers is therefore required.

### Global salt exchanges regulating North Atlantic salinity

**Fig. 3:**
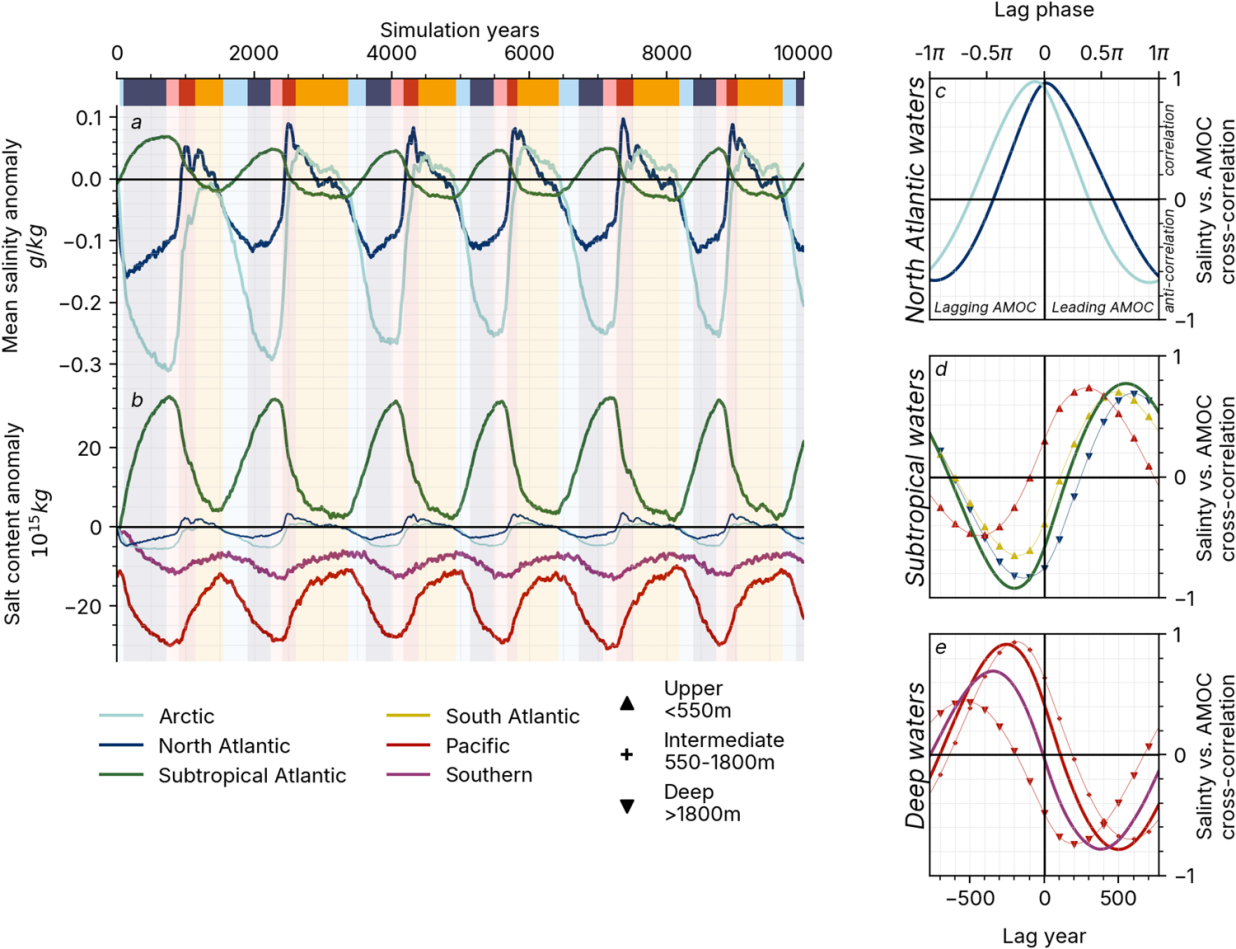
Inter-basin salinity oscillations. Time-series of the *20.7k* simulation anomaly relative to the *CTRL_LGM* simulation for **a** mean salinity and **b** salt content. Background shading indicates the phases defined in Fig. 1. Cross-correlation between the normalised salinity budget anomalies and AMOC index (see Fig. 1a) in **c** the North Atlantic waters, **d** the Subtropical Atlantic waters and **e** the Deep waters domain. The lag phase in the cross-correlations is calculated with the 1540-year periodicity derived in Section S3. For readability purposes, only the signals from the upper and deep Arctic, North Atlantic, subtropical Atlantic, Southern Ocean and Pacific are plotted in the foreground in **c**–**e**, with the other cross-correlations visible in the background

Fluxes of salt in and out of the cluster formed by the North Atlantic and Arctic basins, referred to as the *North Atlantic waters* in the rest of the analysis, can disrupt the stratification of the deep water formation sites. Figure 3a shows that in the *20.7k* simulation, these two regions experience mean salinity shifts larger than in any other basins (Figure S5, the zones used in this paragraph are defined in Figure S1a). Between the *cold* and *warm* phases, the mean salinity changes by around ${0.2\,\textrm{g}.\mathrm{kg^{-1}}}$ in the North Atlantic and ${0.35\,\textrm{g}.\mathrm{kg^{-1}}}$ in the Arctic, reaching up to ${0.75\,\textrm{g}.\mathrm{kg^{-1}}}$ in the North Atlantic and ${1.1\,\textrm{g}.\mathrm{kg^{-1}}}$ in the Arctic if we restrict the domain to the first 550 ms of the water column (Figure S5). Such variations of mean salinity only take small changes in the salinity budgets (Fig. 3b) because of the relatively small volume of these two basins. We observe a strong correlation between the AMOC time series and the salinity content in the North Atlantic waters in Fig. 3c. This implies that the AMOC is the main driver behind salinity changes in the domain. The North Atlantic waters’ salinity reaches its minimum roughly at the same time as the AMOC index ($\sim$ 750 years after its maximum, Fig. 3c), but the North Atlantic waters’ salinity variations are more muted than the AMOC signal during the cold mode.

The subtropical Atlantic mean salinity is ahead of the North Atlantic signal by $\sim$ 500 years (Fig. 3a, d). The correlation and anti-correlation coefficients are around ${0.8\,.}$ and ${-\,0.8\,.}$, respectively, due to less sharp phase transitions in the subtropical Atlantic salinity signal. Despite large changes in the salt budget (Fig. 3b), the mean salinity in the subtropical Atlantic varies by less than ${0.1\,\textrm{g}.\mathrm{kg^{-1}}}$ throughout the simulation (Fig. 3a). The subtropical Atlantic signal is echoed in the deep North Atlantic and the upper South Atlantic, forming another domain that we call the *Subtropical waters*. The upper layer of the Pacific basin follows a similar trend but is delayed by $\sim$ 200 years compared to the *Subtropical waters*. To conserve global salt content (see Sect. 2.2), the large changes of salt content in the *Subtropical waters* have to be compensated for in other basins.

We find changes proportional but of opposite sign to the salinity in the *Subtropical waters* in the Pacific and, to a lower extent, the Southern oceans (Fig. 3b). The salt content anomaly in these basins is dominated by the intermediate and deep water signal (below 550 ms, Figure S6). This domain, which we call the *Deep waters*, shows less spatial coherence as changes in the deep waters (below 1800 ms) experience a delay of $\sim$ 250 years compared to changes in the intermediate waters (e.g. Pacific signal in Fig. 4.2e). Overall, the *deep waters* signal lags behind the AMOC signal by $\sim$ 250 years, and its opposite signal leads the AMOC by $\sim$ 500 years.

In summary, the global salt fluxes resemble a relaxation oscillator where salt is slowly transported from the deep Pacific reservoir to the upper waters of the Atlantic during the *cold* phases, and exported from the Atlantic to the Pacific during the *warm* phases (Fig. 3b). Salt anomalies in the North Atlantic are remarkably coupled to changes in the AMOC strength and follow the *Subtropical* and *Deep* waters salt exchanges with a 500-year delay. The global salt redistribution influences the North Atlantic stratification changes described in Sect. 4.1, and both components come together to explain the mechanisms for the AMOC oscillations that will be described in Sect. 5.

## The convection–advection oscillator mechanism

**Fig. 4:**
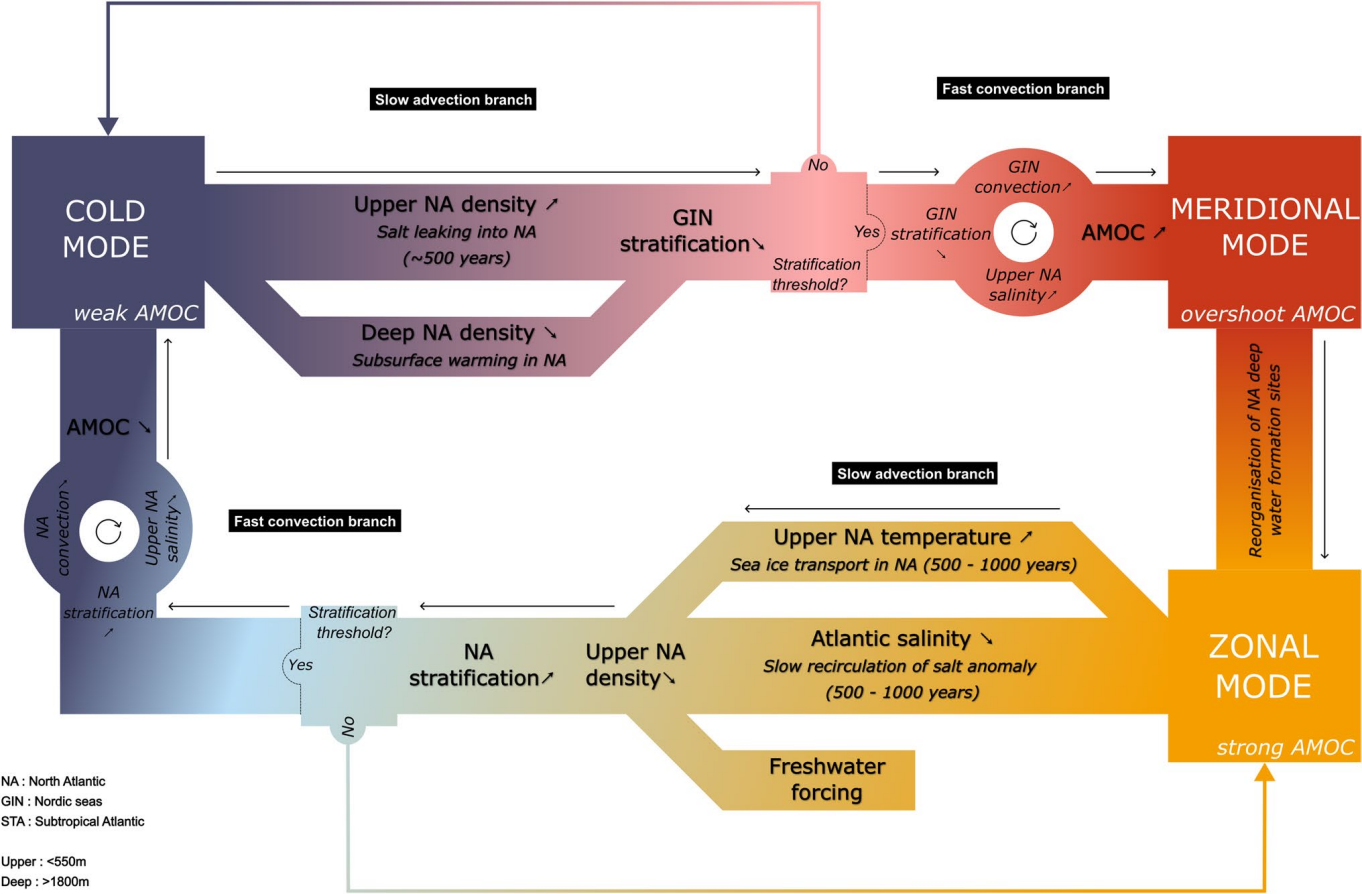
The oscillator mechanism. Millennial-scale variability mechanism observed in *20.7k*. The colours correspond to the phases defined in Fig. 1 to the regions defined in Figure S1

In this section, we introduce the **convection–advection oscillator** mechanism to explain the millennial-scale variability simulated in *20.7k*. This mechanism, summarised in Fig. 4, is based on the fast/slow components coupling framework introduced in (Roberts and Saha 2017). The North Atlantic deep water formation activation in response to stratification thresholds makes up the **fast convection** component, and the large scale reorganisation of global salt content changing buoyancy fluxes in the North Atlantic makes up the **slow advection** component. During *cold* phases, the North Atlantic stratification is high and the AMOC is weak (Fig. 2). Salt accumulation in the subtropical Atlantic (Fig. 3b) leaks into the North Atlantic (Fig. 2p) while the accumulation of subsurface heat decreases the deep water formation sites’ stratification (Fig. 2*p*). In about 500 years, the gradual drop of stratification passes a threshold where the fast convection component is reactivated. There is a positive feedback loop between the strengthening of North Atlantic deep convection and salt advection into the Upper North Atlantic that leads to the abrupt acceleration of the AMOC. After a transition to a *warm* phase, the North Atlantic stratification is low and the AMOC is strong (Fig. 2o, r). The overturning circulation flushes the salt excess to the deep oceans, and the Atlantic becomes progressively salt depleted (Fig. 3b), reducing its upper water density. This slow decrease lasts for 500–1000 years and the southwards transport of sea ice in the North Atlantic (Fig. 2a–c) progressively resumes the subsurface warming (Fig. 2k, l). A more abrupt drop is observed at the start of the *cooling* period when a new threshold is crossed and the fast convection component is reactivated. The positive feedback loop between the decrease of North Atlantic convection and the slowdown of salt import into the North Atlantic leads to the deactivation of the AMOC and the system returns to a *cold* phase.

The **meltwater discharge** creates a fresh surface layer in the North Atlantic that reduces the upper salinity and can lead to deep water formation deactivation when targeted at deep convection sites [see Section 4 by Romé et al. (2022)]. The decrease of upper water salinity triggers an initial shift from the *control zonal* phase to a *cold* phase before the deep waters have time to adapt to the perturbation. This transition happens in the first few hundred years and kickstart the oscillator. It takes two to three cycles until the end of the transient regime where the influence of the freshwater perturbation is observed, for instance, in the deep density profiles in Figure S4). For this reason, we focus the following analysis on the last three cycles of the *oscillating* simulation. In the rest of the run, the constant meltwater input acts to decrease the upper North Atlantic density.

The rest of this analysis relies on the more detailed information gathered from the salinity tendency diagnostics presented in Section S6. In order to reactivate North Atlantic convection, the freshwater forcing needs to be compensated for by salinity fluxes into the North Atlantic basin: this is the role of the **slow advection** component. The subtropical Atlantic is a net evaporation basin and tends to accumulate salt in the absence of the AMOC advective flux (Fig. 3b, Figure S9). This excess of salt is eventually transferred to the upper North Atlantic by gyre circulation and diffusion (Figure S8), increasing the upper North Atlantic density (Fig. 2p–r, Figure S12). When the AMOC is reactivated, the salt excess is progressively evacuated from the upper North Atlantic to the deep ocean through convection (Fig. 3e, Figure S4), and then redistributed along the oceanic pathway. The global salinity transfer can be seen as a salinity anomaly slowly moving between the subtropical Atlantic and the Pacific (Weijer and Dijkstra 2003), and it takes more than a thousand years for the waters for the global salinity exchanges to reach a new equilibrium state (Fig. 3b).

The combination of salt fluxes entering the North Atlantic, subsurface warming and sea ice transport modify stratification at the deep water formation sites and trigger the **fast convection component**. During the *cold* phases, the insulating effect of sea ice described by Marcott et al. (2011) led to a warming and density drop of the entire water column (Figure S12). This effect is the most prominent at high latitudes, including the GIN Seas, where the sea ice is perennial (Fig. 2a, p). In the upper North Atlantic waters, the sea ice effect is superseded by the salinity input from the subtropical Atlantic (Figure S12a–c). In the GIN Seas, a vertical stratification is eventually crossed leading to the reactivation of deep convection in this region. The salt advection feedback (Drijfhout et al. 2015; Weijer et al. 2019)accelerate this transition into a *warming* phase.

During the *warm* phases, the decreasing salt availability in the subtropical Atlantic reduces upper North Atlantic water density (Figure S8), and the slow evacuation of subsurface heat accumulated in the Irminger and Iceland basins during the cold phase increases deep waters density (Figure S13). Despite the gradually expanding sea ice in the North Atlantic, the absence of a clear salinity signal in the region that would indicate the brine rejection (Figure S8) indicate that the sea ice is more likely to be transported in rather than formed in the regions. Instead, further reduction of upper water density (Fig. 2p–r) can be linked to an increase of temperatures resulting from the insulating effect of sea ice (Figure S13d–h). As the stratification increases, it crosses a threshold where the positive feedback leads to a sharp decline of the AMOC.

## Sensitivity of the convection–advection mechanism to climate conditions

**Fig. 5:**
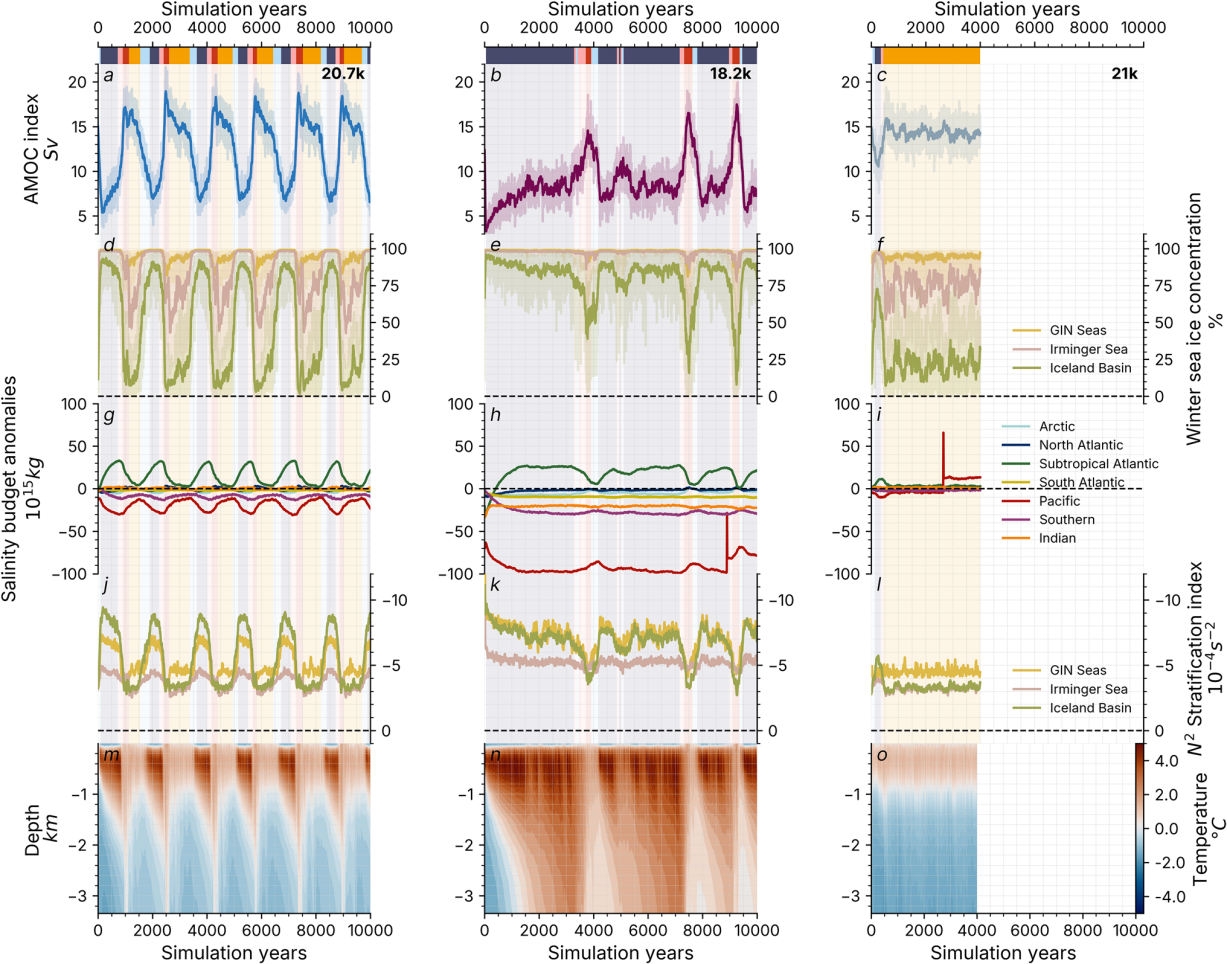
Comparison to the non-oscillating simulations. 30-year running means (solid lines) and annual mean (transparent lines) time series for **a**–**c** maximum AMOC strength, **d**–**f** winter sea ice concentration, **g**–**i** salinity budgets in global ocean basins (see Fig. 3a), **j**–**l** N2 stratification index (Li et al. 2020) in the deep water formation sites (low negative values indicate high stratification) and **m**–**o** ocean potential temperature profiles in the North Atlantic for three meltwater snapshot simulations (see Table 1). The abrupt increase of Pacific salinity in panels **j** and **k** correspond to a change of the basin volume introduced by the bathymetry smoothing algorithm in the Philippines described by Romé et al. (2022)

To gain insight into the range of climate conditions under which the convection–advection oscillator mechanism (Sect. 5) is active, we extend our analysis to the additional glacial meltwater experiments. A comparison of the dynamics of the main simulations in this article is given in Fig. 5.

The magnitude of the freshwater perturbation is critical for sustaining the oscillations. In the *21k* simulation, a weaker freshwater forcing compared to the *20.7k* simulation does not significantly alter the global salt distribution (Fig. 5i). The AMOC index only drops by ${5\,\textrm{Sv}}$ (Fig. 5c) and the salinity anomaly is dispersed before a stratification threshold is crossed in the North Atlantic (Fig. 5l). The *21k* simulation eventually recovers to a *zonal* steady state, the sea ice edge settles after another 500 years (Fig. 5f), and the simulation shows no further signs of instability. The *warm steady state* simulations, such as the *21k* simulation, behave like an over-damped oscillator: the applied forcing transiently kicks the system out of the initial state, but the system returns to a warm state after less than a thousand years. This suggests that the zonal state retains its stability and that the simulation remains in its basin of attraction following the onset of the forcing.

In the *18.2k* simulation, a strong meltwater input triggers a near-collapse of the AMOC and high North Atlantic stratification (Fig. 5k) prevents the recovery of deep water formation. It takes about 1000 years for the global salinity distribution to reach a new equilibrium (Fig. 5h), without any stratification threshold being crossed that could reinvigorate the AMOC. In the case of the *18.2k* simulation, there are short-lived excursions from a stable *cold* state to a transient *meridional* state, reminiscent of *excitable* behaviour. The *cold* simulations are therefore defined as *cold excitable steady state* simulation with the *cold* mode acting as an attractor. As an alternative to multistable, oscillatory or resonant dynamics, Riechers et al. (2024) conducted a study where an excitable monostable model can capture the shape of D–O cycles. In our case, the *18.2k* simulation does not resemble D–O variability because of short warm modes and the absence of slow cooling. The AMOC recoveries result from the warming of the North Atlantic water column visible in Fig. 5n, fuelled by sea ice accumulation over the deep water formation sites (Fig. 5e). The recoveries are strictly confined to the GIN Seas, and the stratification of the Irminger Sea remains too high to transition into a *zonal* steady state. In other words, the salinity import from the subtropical Atlantic is not strong enough to compensate for the freshwater forcing and, therefore, to trigger the positive feedback that would reactivate the AMOC. Although the excursions into a *meridional* mode occur at what seems to be random intervals, their frequency increases over time due to continuous subsurface heat accumulation, indicating that the asymptotic state may not have been reached.

In between the *warm steady state* and the *cold excitable steady state*, the *oscillating* simulations are obtained in a small window of opportunity where the freshwater forcing is strong enough to trigger a *cold* mode, but not too strong to prevent a subsequent transition to one of the *warm* modes. In this window of opportunity, the solution is attracted to a chaotic limit cycle (closed curve in the state space) around which it evolves quasi-periodically. Possibly, the warm mode undergoes a supercritical Hopf bifurcation as the freshwater input is increased at the relevant locations, giving rise to stable oscillations (Boers et al. 2022). Finally, small changes in meltwater discharge can modify the shape and periodicity of the oscillations described by Romé et al. (2022), but do not change the underlying mechanism.

## The scope of application of the convection–advection oscillator mechanism

The *convection–advection* oscillator mechanism presented here was identified in the *20.7k* simulation introduced in Romé et al. (2022), and its relevance may depend on the simulation inputs, the model settings and the choice of the model. The comparison to Armstrong et al. (2022)’s simulations, using the same version of the model, indicates that several mechanisms for millennial-scale variability can coexist within one model. Armstrong et al. (2022) use the 30 ka BP background climate in which the Irminger Sea and European coast convection sites are still active during the cold modes, and they can trigger spontaneous oscillations in the absence of additional freshwater. The *convection–advection* oscillator should be seen as a new occurrence of a *fast-slow* oscillator (see Figure S15) based on the concept of a relaxation oscillator (Pikovsky et al. 2001; Crucifix 2012; Roberts and Saha 2017). Although its specific interplay of fast and slow processes is new, the building blocks of the mechanism have been identified in former studies. For example, Klockmann et al. (2020) and Vettoretti and Peltier (2018) highlighted the role of the subtropical Atlantic salt accumulation and depletion. The difference of time response between the upper and lower waters in the North Atlantic as a driver of instability is central to the deep-decoupling oscillations theory (Winton and Sarachik 1993), and observed in Vettoretti and Peltier (2018) and Kuniyoshi et al. (2022). The hypothesis that salt oscillations can reach further than the Atlantic basin was introduced in Weijer and Dijkstra (2003). The insulating effect of sea ice is similar to the one described by Kuniyoshi et al. (2022). Finally, the response of the North Atlantic convection to changes in stratification fits well with the description in Vettoretti and Peltier (2018).

On the other hand, our *convection–advection* oscillator does not include previously proposed *slow* components such as changes of Southern Ocean density (e.g., Oka et al. 2021; Vettoretti et al. 2022). These changes can modify the Atlantic stratification in models with a strong sensitivity of the AABW to the Southern Ocean dynamics, but in the *20.7k* simulation the small density increase and decrease due to the shrinking and expansion of the AABW during the cold and warm phases, respectively, are too weak to significantly impact the North Atlantic convection (Figure S14). Arctic sea ice transport (e.g., Vettoretti and Peltier 2018) is at play in our simulations, but sea ice formation and melting appear to play a lesser role than inter-basin salt transport in regards to the North Atlantic salinity changes, contrary to Vettoretti and Peltier (2018) or Armstrong et al. (2022). Armstrong et al. (2022) also identify large changes of precipitation and evaporation in the Atlantic as a driver of the salt oscillator. This tendency is not dominant in our simulations (Fig. S8). The *fast* components controlling the reactivation of deep water formation sites can also be the result of different physical processes in different experiments. Klockmann et al. (2020) and Armstrong et al. (2022) discuss the role of wind driven-feedback in the abrupt re(de)activation of deep water formation. There is no clear evidence of a similar phenomenon happening in our simulations. Additionally, both Vettoretti and Peltier (2016) and Kuniyoshi et al. (2022), associate the overshoot phase with polynyas opening in the sea-ice-covered Irminger Sea, a phenomenon that is not observed in any of our simulations. Finally, the regularity of the oscillations indicates a weak role of stochastic forcing in *20.7k* (Figure S3), but may play a major role in *18.2k*.

Another peculiarity of our oscillating simulations is the existence of two warm states and the transition from a *meridional* to a *zonal* phase. Cheng et al. (2011) simulated a similar two-step warming phase, but in their case the abrupt recovery of deep water formation was observed first in the Labrador Sea and then in the GIN Seas. In this simulation, the transition is smooth and the *meridional* phase is akin to an **overshoot phase** of the *zonal* phase, a phenomenon regularly simulated in GCM millennial-scale variability (Peltier and Vettoretti 2014; Kuniyoshi et al. 2022; Vettoretti et al. 2022, e.g.,). It happens when deep water formation shifts from the GIN Seas to the Irminger Sea due to local reorganisation of convection and gyre circulation (Figure S8). The *meridional* phase witnes a drop in the advective flux of salt into the North Atlantic (Figure S8b, d) that leads to a negative salinity import into the basin (Figure S8c), making this phase unstable. While the transition from a *meridional* to a *zonal* phase is not an essential part of the overarching convection–advection mechanism, it may play a more central role in other simulations using different experiment configurations. It is possible that, for instance, longer *meridional* phases can be sustained in simulations where the salinity tendency inversion in the North Atlantic is not obtained, such as in the *17.8k* simulation in Romé et al. (2022).

The combination of fast and slow components involved in millennial-scale variability implies a multiplicity of possible mechanisms, probably leading to different shapes, duration and regularity of the oscillations. In *20.7k*, the pace of the simulations is set by two limiting processes. During the *cold* phase, it takes about 500 years for a sufficient amount of salt to leak from the subtropical Atlantic into the North Atlantic in order to cross a North Atlantic stratification threshold. In the *warm* phases, the salt anomaly takes more than a thousand years to travel across the entire track of the overturning circulation to reach the Subtropical Atlantic. These two processes are dependent on model parametrisation and input fields. Other factors impacting the emergence of sustained oscillations could be associated with slow components such as the rate of Arctic sea ice transport (Vettoretti and Peltier 2018) or the bipolar see-saw (Crowley 1992; Stocker 1998).

In Sect. 5, we showed that the convection–advection mechanism requires the propagation of a salinity anomaly between the ocean basins. One of the main parameters that can impact global salt distribution is the choice of the salinity conservation scheme. In our simulations, the ice sheet extent does not vary with the meltwater discharge, and therefore requires the use of a global salinity conservation algorithm to prevent salinity drifts (see Sect. 2.2 Dentith et al. 2019). In order to test the effect of the salinity conservation scheme on the convection–advection mechanism, we ran a simulation without any salinity conservation: the *20.7k_no_gst* simulation. This simulation follows the evolution of the *20.7k* simulation for the first 500 years (Figure S16a, b) before the salt distribution becomes unstable at the end of the *cold* phase: the salinity of all the oceans then increases in every basin during the *zonal* phase (Figure S16c, d). In this configuration, the tropical Atlantic salt content is in excess, and the North Atlantic upper density remains high, maintaining active deep-water formation (Figure S16f), pushing the system away from the window of opportunity. Salt accumulation may seem counter-intuitive in a freshwater hosing experiment, but the rigid lid parametrisation prevents the model from fully resolving the water cycle (Demory et al. 2014), which can lead to these unrealistic drifts. In addition to its role in closing the water budget, the applied salinity correction is a way to emulate the stabilising power of dynamical ice sheets on global salinity. This phenomenon could play an active role in the occurrence of millennial-scale variability: Wickert et al. (2023), for instance, proposed a mechanism where the storage and release of freshwater in the Laurentide ice sheet during the stadials and interstadials, respectively, can provide a negative feedback that impedes the deglaciation of the ice sheet. On the other hand, there are certain times during the last glacial period when the meltwater released by the destabilisation of the ice sheets, such as during Heinrich events (Menviel et al. 2014, 2020) or saddle collapse (Gregoire et al. 2012; Ivanovic et al. 2017), has acted as a driver for abrupt environmental change. This makes the case for further coupled-climate ice sheet experiments (e.g., Alvarez-Solas et al. 2019; Schannwell et al. 2024; Mikolajewicz et al. 2024 in order to include prognostic feedbacks from the ice sheets and better constrain the freshwater forcing during the last glacial period and deglaciation.

## Conclusion

We presented a mechanism explaining the millennial-scale variability observed in a last glacial maximum HadCM3 general circulation model simulation, forced with a constant pattern of deglacial meltwater. The simulation displays a periodic evolution along a limit cycle through a *cold* phase—where the AMOC is weak and there is no deep water formation in the North Atlantic, a *meridional warm* phase—where the AMOC is strong and convection predominates in the Nordic seas, and a *zonal warm* phase—where the AMOC is strong and convection shifts primarily to the Irminger Sea.

The convection–advection oscillator is composed of a *slow advection* component and a *fast convection* component, with the AMOC coupling the two. The *slow advection* component transports a salt anomaly between the subtropical Atlantic and mainly the deep Pacific. The *fast convection* component responds to thresholds in North Atlantic stratification by reactivating or deactivating deep water formation sites, amplified by the positive salt-advection feedback. During *cold* phases, the salt accumulates in the subtropical Atlantic and slowly leaks into the North Atlantic to increase its upper-ocean density. In addition, progressive subsurface warming decreases the deep density in sea ice-covered regions. This leads to a decrease in the stratification of the Nordic Seas until reaching a threshold where convection rapidly reactivates. The resulting *meridional* phase acts as an overshoot mode before deep water formation shifts to a predominantly *zonal* configuration. During the *warm* phases, the Atlantic becomes depleted in salt because of the slow circulation of the subtropical salt anomaly along the deep ocean pathway. In the North Atlantic, the subsurface heat accumulated during *cold* phases is progressively being evacuated, while the upper waters remain warm due to southwards sea ice transport into the North Atlantic. The salt and temperature changes lead to a slow increase in the stratification of the Irminger Sea and Iceland Basin. The *cold* mode ends when deep convection reaches a threshold of deactivation, leading to a rapid weakening of the AMOC driven by the salt-advection feedback.

Starting from the AMOC control state, the model is forced with the respective deglacial freshwater pattern at the beginning of each simulation. This perturbation has to be strong enough to deactivate the North Atlantic deep water formation sites, but not too strong to allow for the crossing of a threshold from a *cold* mode back into a *warm* mode within the span of the global salt redistribution. In the absence of salinity correction, the global salinity anomaly between the Pacific and Subtropical Atlantic is superseded by a global salinity drift, leading to an abnormally high upper North Atlantic density and moving the system out of the window of opportunity.

The simulations analysed in the paper demonstrate the concept of a window of opportunity, associated with meltwater discharge. Depending on the forcing, we observe a *warm steady state* simulation where the system sustains North Atlantic deep water formation despite the freshwater input, a *limit cycle* simulation where the system follows the convection–advection oscillator mechanism, and an *excitable cold steady state* simulation, where a persistence in the weak AMOC state is interrupted by short-lived recoveries of North Atlantic convection. Further sensitivity experiments involving a more comprehensive range of boundary conditions (such as atmospheric greenhouse gas concentrations, orbital parameters, ice sheet geometry, salinity conservation scheme), climate forcing and physical parameter settings are needed to categorically constrain the regions in parameter space where these different behaviours occur.

While model and boundary condition dependencies influence the results in any study of simulated abrupt climate change, we are at an exciting juncture where common patterns between the different mechanisms presented across the literature are starting to crystallise. This study evidences how concepts of dynamical systems theory can guide the analysis of model simulations towards a unified understanding of millennial-scale climate variability. Finally, in the absence of dynamical ice sheets, salinity conservation is a necessary condition for obtaining millennial-scale oscillations in our simulations, and the introduction of climate-ice sheet feedback would represent a significant step towards simulations of more realistic millennial-scale variability.

## Supplementary Information

Below is the link to the electronic supplementary material.

Supplementary file 1 (pdf 7369 KB)

## Data Availability

The data used to produce the Figures of this article are available on the CEDA archive under Romé et al. (2024). Additional data can be requested to the corresponding author.

## Code availability

The code used to produce the Figures of this article is available on Zenodo under Romé (2024).

## Declarations

### Prior

A prior version of this manuscript was published in YMR’s PhD thesis and can be accessed at https://etheses.whiterose.ac.uk/34761/.
