# Supplementary material for: Simulated millennial-scale climate variability driven by a convection–advection oscillator
Source: Clim Dyn. 2025 Mar 7;63(3):150. doi: 10.1007/s00382-025-07630-x (PMC11885369; doi:10.1007/s00382-025-07630-x)
Supplement: Supplementary file 1 — (pdf 7369 KB) [file 382_2025_7630_MOESM1_ESM.pdf]

Supplementary information: Simulated glacial millennial-scale variability driven by a coupled inter-basin salt oscillator

Yvan M. Romé[1*], Ruza F. Ivanovic[1], Lauren J. Gregoire[1], Didier Swingedouw[2], Sam Sherriff-Tadano[3], Reyk Börner[4]

[1]School of Earth and Environment, University of Leeds, Woodhouse Lane, Leeds, LS2 9JT, United Kingdom.
[2]Univ. Bordeaux, CNRS, Bordeaux INP, EPOC, UMR 5805, Pessac, 33600, France.
[3]University of the Ryukyus, Faculty of Science, 1 Senbaru, Nishihara, Nakagami District Okinawa, 903-0129, Japan.
[4]Department of Mathematics and Statistics, University of Reading, Whiteknights House, Reading, RG6 6UR, United Kingdom.

*Corresponding author(s). E-mail(s): eeymr@leeds.ac.uk;
Contributing authors: r.ivanovice@leeds.ac.uk; l.j.gregoire@leeds.ac.uk;

Contents of this file

1. Text S1 to S8
2. Figures S1 to S16

Introduction

The supporting information presented here contains eight sections and sixteen figures as supplements to the main text. This includes details on the zone definitions (Section S1, Figure S1), an expanded version of the anatomy of the oscillations Figure introduced in Figure 1 only for the *20.7k* simulation (Section S2, Figure S2), a frequency analysis of the oscillating simulations (Section S3, Figure S3), an expanded version of the North Atlantic ocean profiles in the deep water formation sites used to plot Figure 2 (Section S4, S4), a comprehensive salinity exchange visualisation (Section S5) by plotting the salt budget time series (Figure S6) and the salinity means time series (Figure S5), the salinity tendencies analysis (Section S6) by plotting the salinity tendencies in the ocean basins introduce in Figure S1 (Figure S8 to Figure S11), additional figures used to explain the convection-advection mechanism in Section 5 (Section S7) including the vertical ocean profiles in the deep water formation sites during the *warming* phase (Figure S12) and the *cooling* phase (Figure S13), the evolution of the AABW (Figure S14) and a global concept for millennial-scale variability (Figure S15), and finally a demonstration of the role of the global mean salinity conservation algorithm (Section S8, Figure S16).

S1 Zone definitions

The geographical zones used throughout the article to identify different ocean basins or sub-basins were defined manually over the HadCM3 ocean grid. The global ocean basins and the North Atlantic regions are plotted in Figure S1.

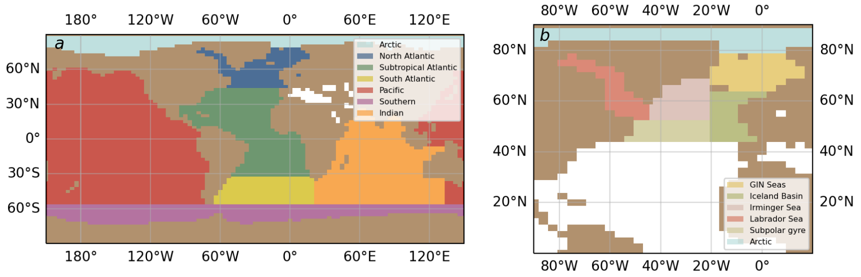

Fig. S1 Zone definitions. Definition of the zones in the global(a) and North Atlantic (b) regions.

S2 Anatomy of all of the simulations

The anatomy of the *20.7k* simulation was plotted in Figure 1. In Figure S2, we reproduce this figure for the simulations introduced in Table 1. In this Figure, we use the same terminology and visual markers than in Section 3, but the locations of the spans in the North Atlantic mixed layer depth state space was to be adapted to each individual simulation.

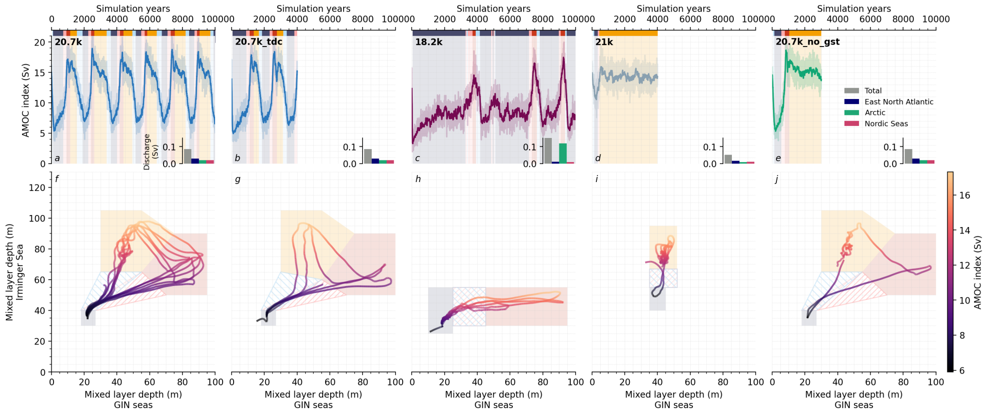

Fig. S2 Anatomy of the simulations expanded. As for 1 but for the *20.7k* (*a,f*), *20.7k_tdc* (*b,g*), *18.2k* (*c,h*), *21k* (*d,i*) and *20.7k_no_gst* (*e,j*) simulations.

S3 Frequency analysis of the oscillating simulations

In order to identify the oscillating simulations and to derive their associated natural frequency, we can adapt the frequency analysis performed by Romé et al (2022). This simple algorithm uses the Scilab Python tools to undertake a Fourier filtering of the raw AMOC-index time series data (taken as the maximum strength of the overturning circulation at 26.5° N). A peak in the harmonics of the Fourier distribution indicates the frequency (i.e. periodicity) of an oscillating mode. In the *oscillating* simulations, this peak is around 1,540 years, which was used to calculate the cross-correlation phase in Figure 3. It is also the basis for our use of the low-pass filter centred around $2.10^{-3} yr^{-1}$ for the state space in Figures 1 and S2. For more details about the algorithm, please read Section S5 in Romé et al (2022).

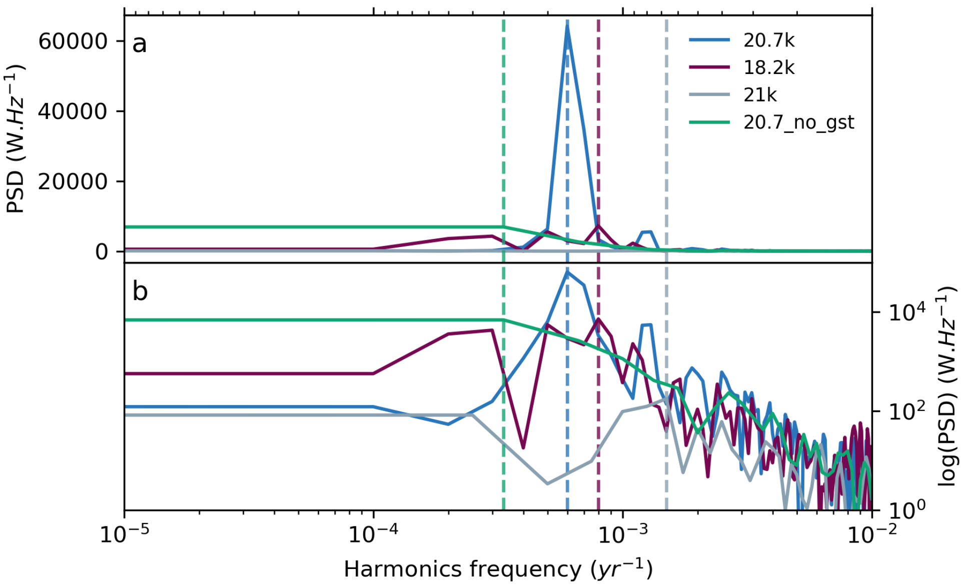

Fig. S3 Spectral analysis. (*a*) Power Spectral Density (PSD, left hand scale) of the unfiltered signal. Dotted lines indicate the dominant frequency/period for each simulation. (*b*) Same as *a* but showing the logarithm of the PSD.

S4 North Atlantic oceanic vertical profiles at deep water formation sites during abrupt climate changes

The temperature, salinity and density profiles of the North Atlantic deep water formation sites that have been used to produce Figure 2, alongside the vertical profiles in the Labrador Sea and Arctic Ocean, are shown in Figure S4. For better readability, only the last 5000 years of the *20.7k* simulation, corresponding roughly to the last three oscillatory cycles, are plotted in this Figure. The spans correspond to Figure 1.

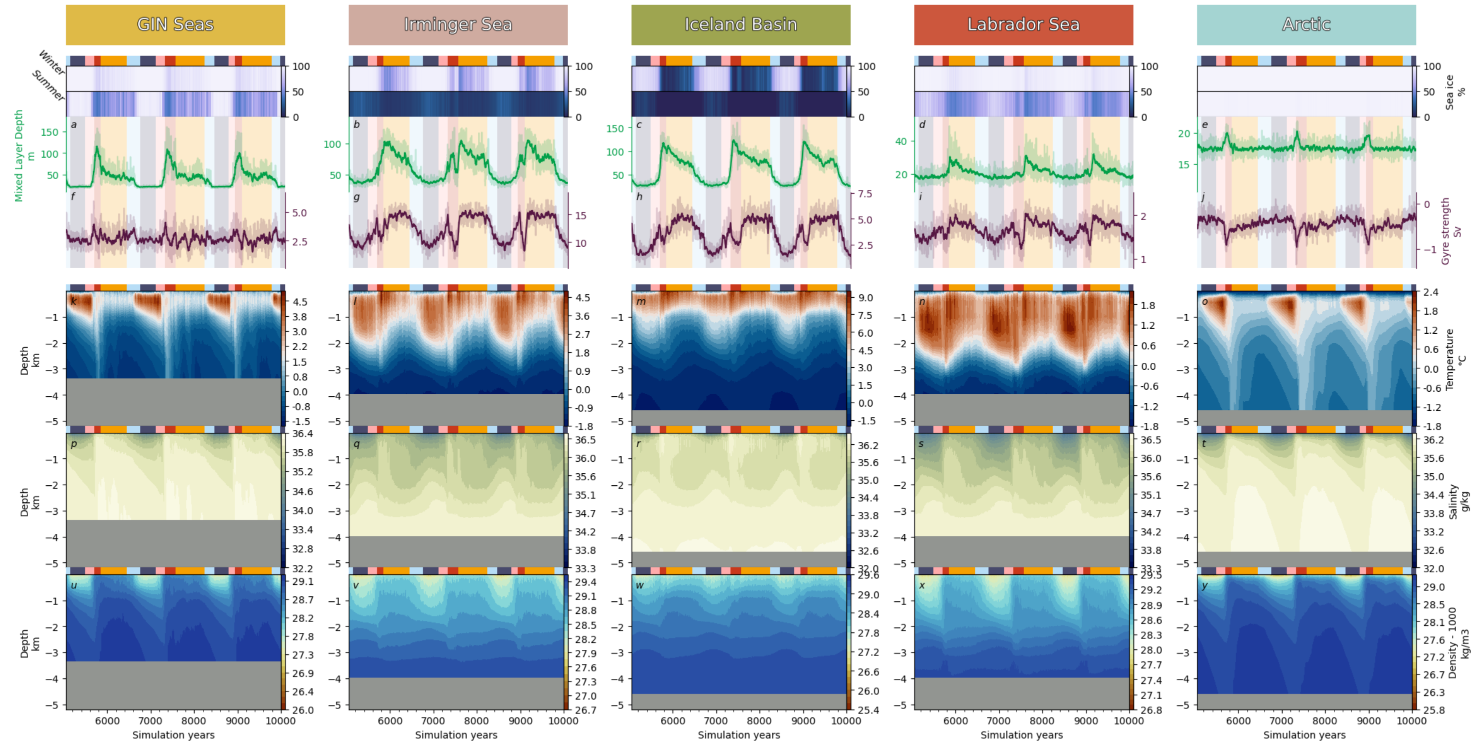

Fig. S4 North Atlantic dynamics at key deep water formation sites, expanded. Similar to Figure 2 for *20.7k*, but including the temperature and salinity profiles and the Labrador Sea and Arctic Ocean (see Figure S1*b*). The time series were expanded to cover the whole experiment.

S5 Salinity means and budget in the global ocean basins

As a supplement to Figure 3, the mean salinity and salinity budget anomalies of the global ocean basins defined in Figure S1 at all three depth bins (*Upper*, *Intermediate* and *Deep* waters) are plotted in Figure S5 and Figure S6, respectively.

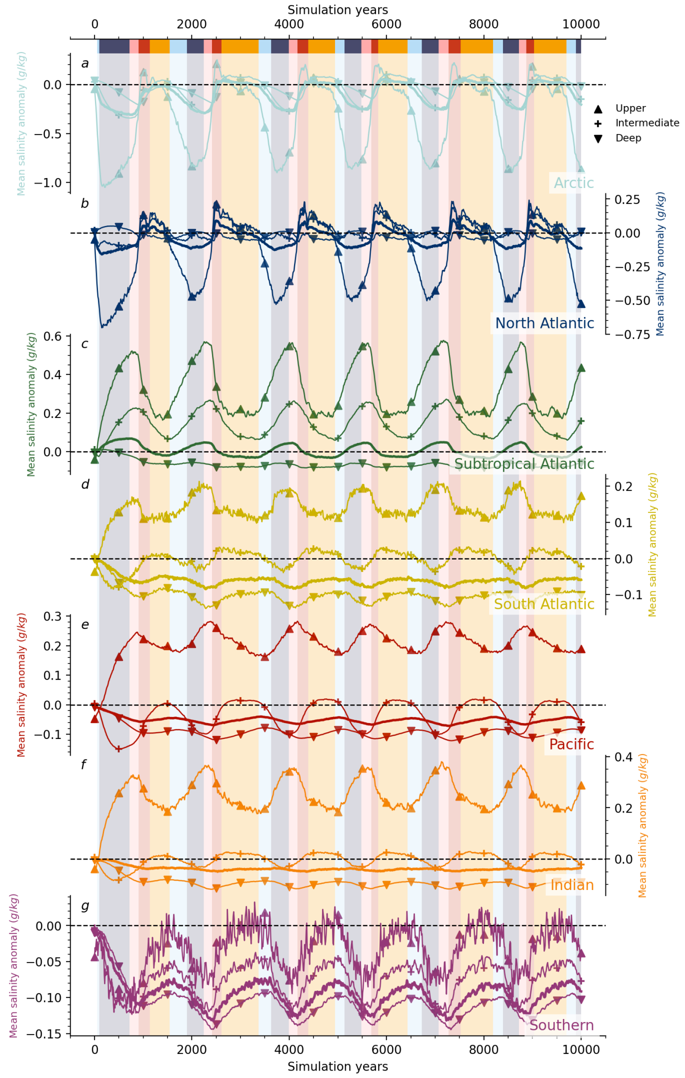

Fig. S5 Global salinity means. As in Figure 3*b*, but for the different basins (see Figure S1*a*), including the upper, intermediate and deep waters.

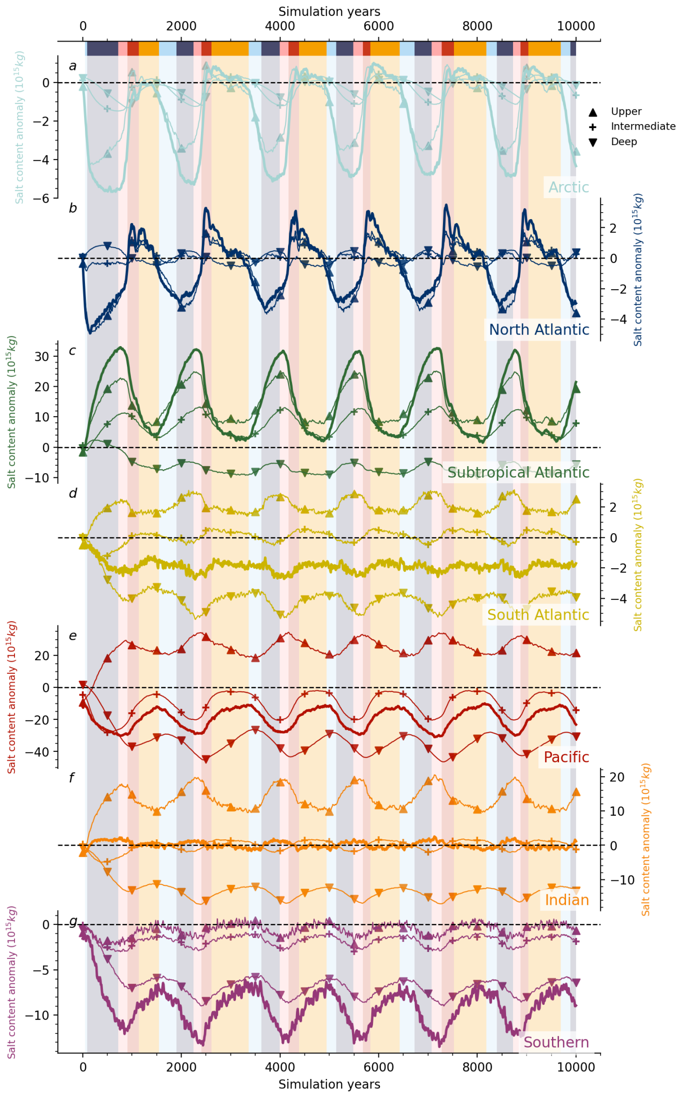

Fig. S6 Global salt content budgets. As in Figure 3*a* in the different basins (see Figure S1*a*), including the upper, intermediate and deep waters

S6 Salinity tendencies diagnostic

The HadCM3 salinity tendency diagnostics were described by Armstrong et al (2022) as a tool to identify the different components of salinity fluxes between ocean grid cells. At each time step and for each ocean grid cell, all the individual processes adding or removing salt are recorded explicitly alongside the net salinity changes. They are clustered into six fields. The *advection* field captures the salinity changes associated with horizontal and vertical water motion coming in and out of the cell. The *diffusion* field captures the sub-grid scale mixing processes, including turbulent mixing and the eddy-induced isopycnal transport, also known as the Gent and McWilliams diffusion (Gent and McWilliams, 1990). The *convection* field covers salinity changes due to vertical convective mixing, including the effect of the mixed layer. The *surface* field contains the salinity changes due to precipitation, evaporation, freshwater input and river run-off. The *ice* field comprises brine rejection and sea ice melting. Finally, the *mediterranean* field captures the salt exchanges coming from the Mediterranean outflow pipe (Ivanovic et al, 2013, 2014). This field was only included in the subtropical Atlantic tendencies in Figure S9. The global salinity correction described in Section 2.2 is not included in the tendencies diagnostic.

In the following section, we plotted the tendencies of the basins introduced in Section 4.2 and defined in Figure S1 for *20.7k_tdc*. They are shown for the Arctic in Figure S7, the North Atlantic in Figure S8, the Subtropical Atlantic in Figure S9, the Pacific in Figure S10. The Indian and South Atlantic basins do not show as clear a signal as the other basins and were omitted for this study.

During the *cold* phases, the North Atlantic salinity tendencies are characterised by the competition between a steady freshwater flux at the surface due to the high precipitation, sea ice melting and meltwater discharge, and advection and diffusion bringing salt into the basin (Figure S8). In the upper waters, the advective and convective fluxes dominate slightly, leading to a slow salt accumulation. The salt is imported

from the subtropical Atlantic, where a strong evaporation-driven salt accumulation is not sufficiently evacuated by a weak AMOC (Figure S9). The salinity decrease in the Pacific is mostly due to a strong net negative salt advection at intermediate and deep depths (Figure S10).

At the start of the *meridional* mode, the resumption of the AMOC translates into a strong salt advection in the North Atlantic. This leads to a salinity increase and the salt excess is redistributed over the entire water column by convection (Figure S8). A similar advection-driven salinity increase is observed in the Arctic basin (Figure S7). This advective flux into the North Atlantic accelerates the salt removal in the subtropical Atlantic, leading to the salt depletion of the basin (Figure S9). Note that the AMOC changes during the *meridional* to *zonal* transition are reflected in the variations of the advective fluxes of both basins. The resumption of the AMOC progressively increase the salt advected in the Pacific (Figure S10).

At the end of the *zonal* phase, the slow decrease of salt advected out of the subtropical Atlantic (Figure S9) leads to a reduction of the North Atlantic salinity and a reduction of its convection (Figure S8). The Arctic also sees its salinity decrease due to the reduction in advective import of salt (Figure S7). When the AMOC is deactivated during the *cooling* phase, the surface fluxes dominate the salinity tendencies in the North Atlantic and in the Arctic accelerating the salinity decrease (Figures S8, Figure S7)). Salt accumulation due to evaporation resumes in the subtropical Atlantic (Figure S9), as well as salt depletion in the Pacific due to a net advective salt export (Figure S10).

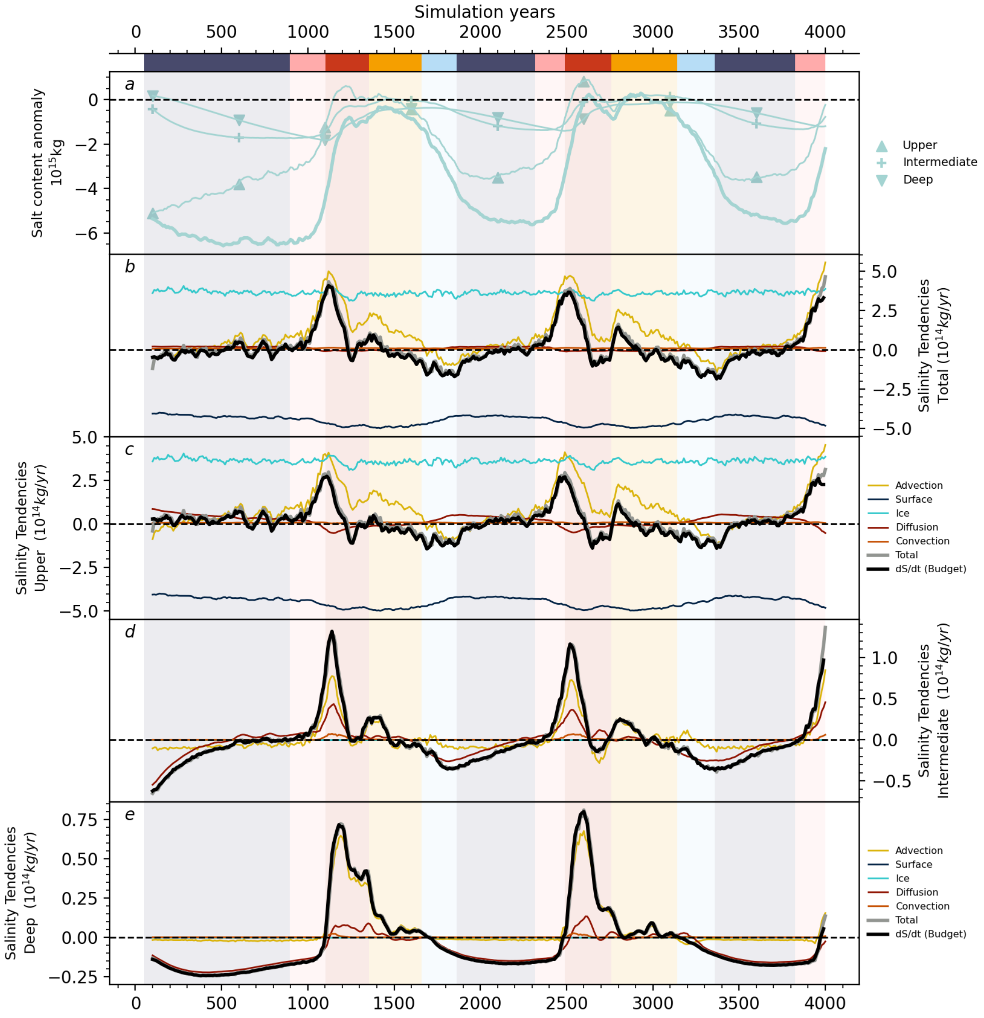

Fig. S7 Salinity tendencies in the Arctic. (a) Salt content anomaly for the upper, intermediate and deep waters in the **Arctic** (see Figure S1). (b-e) 50-year running means of the absolute salinity tendencies in the region for the entire water column (b), the upper waters (c), the intermediate waters (d) and the deep waters (h,i). The detail of the tendency fields is given in Section S6. The field *total* corresponds to the sum of all tendencies, and the field dS/dt corresponds to the salt content variations calculated in HadCM3.

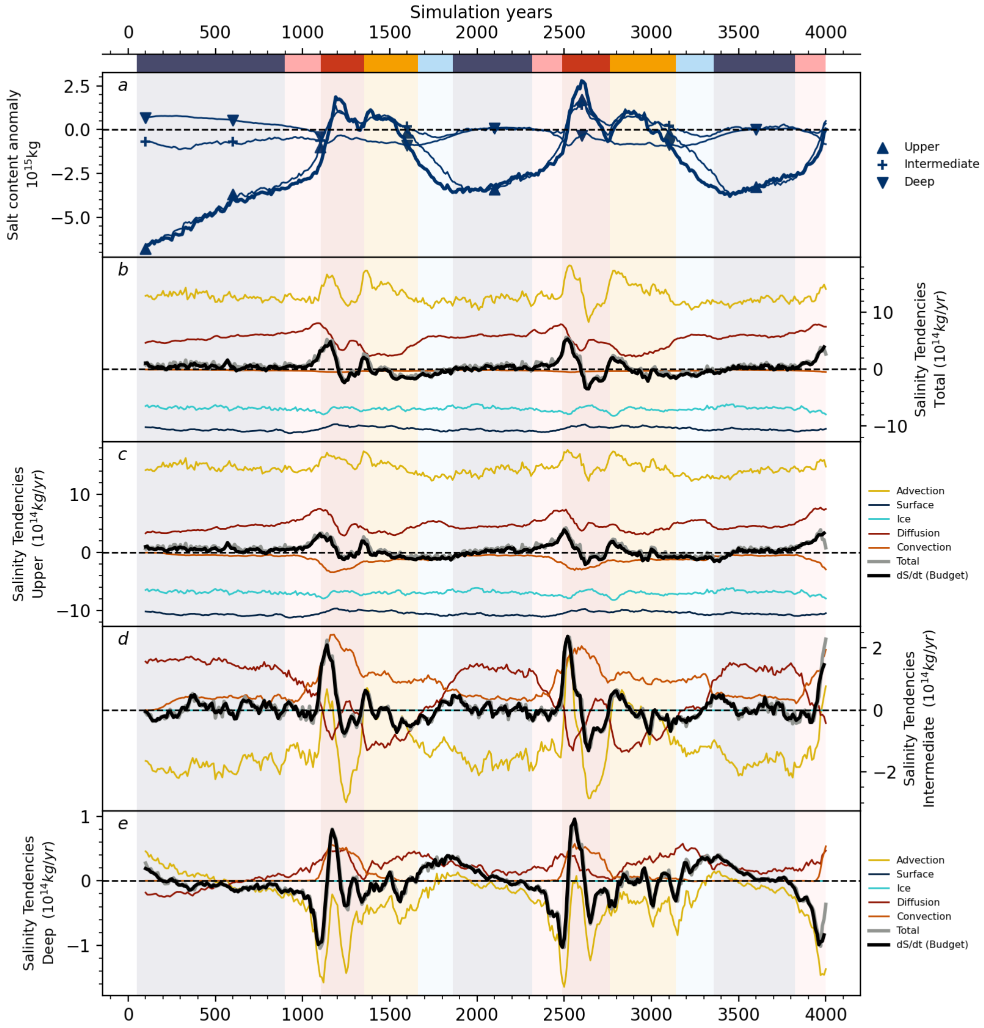

Fig. S8 Salinity tendencies in the North Atlantic. (a) Salt content anomaly for the upper, intermediate and deep waters in the **North Atlantuc** (see Figure S1). (b-e) 50-year running means of the absolute salinity tendencies in the region for the entire water column (b), the upper waters (c), the intermediate waters (d) and the deep waters (e). The detail of the tendency fields is given in Section S6. The field *total* corresponds to the sum of all tendencies, and the field dS/dt corresponds to the salt content variations calculated in HadCM3.

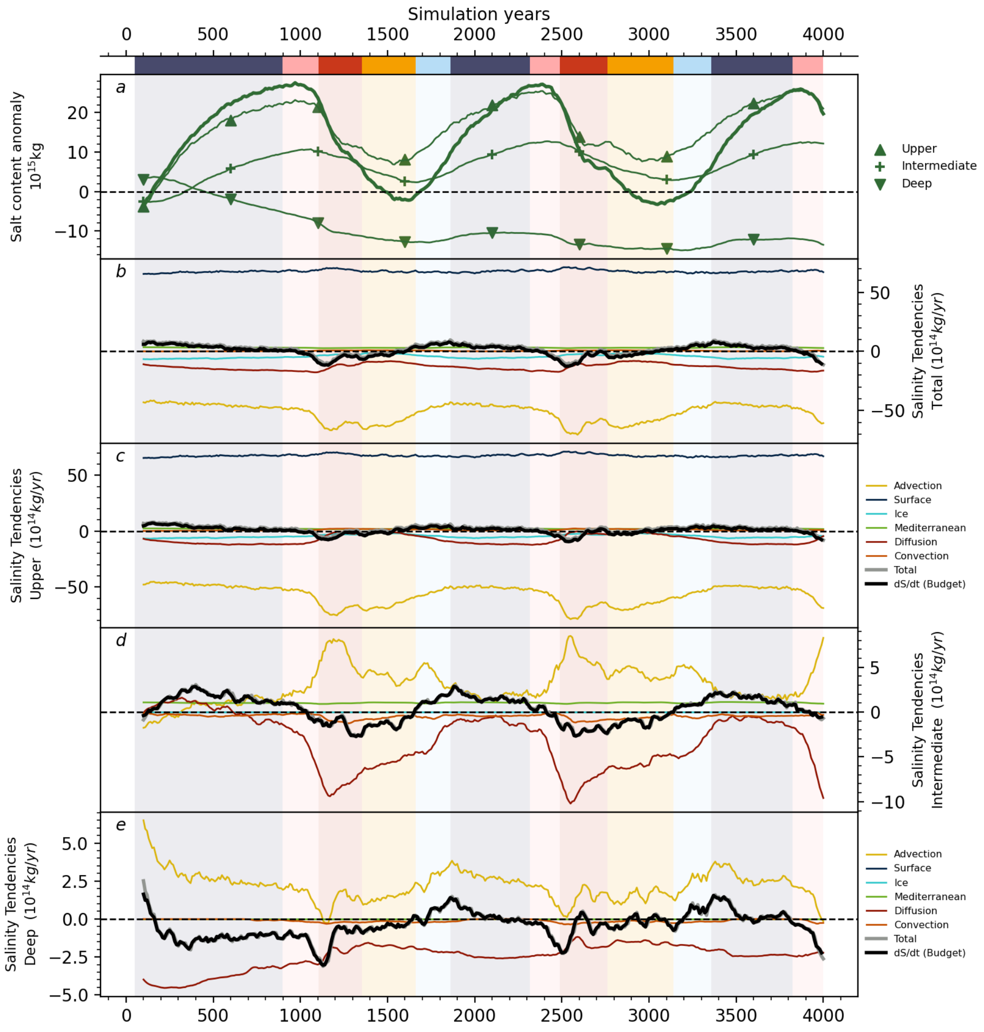

Fig. S9 Salinity tendencies in the subtropical Atlantic. (*a*) Salt content anomaly for the upper, intermediate and deep waters in the **subtropical Atlantic** (see Figure S1). (*b*-*e*) 50-year running means of the absolute salinity tendencies in the region for the entire water column (*b*), the upper waters (*c*), the intermediate waters (*d*) and the deep waters (*e*). The detail of the tendency fields is given in Section S6. The field *total* corresponds to the sum of all tendencies, and the field *dS/dt* corresponds to the salt content variations calculated in HadCM3.

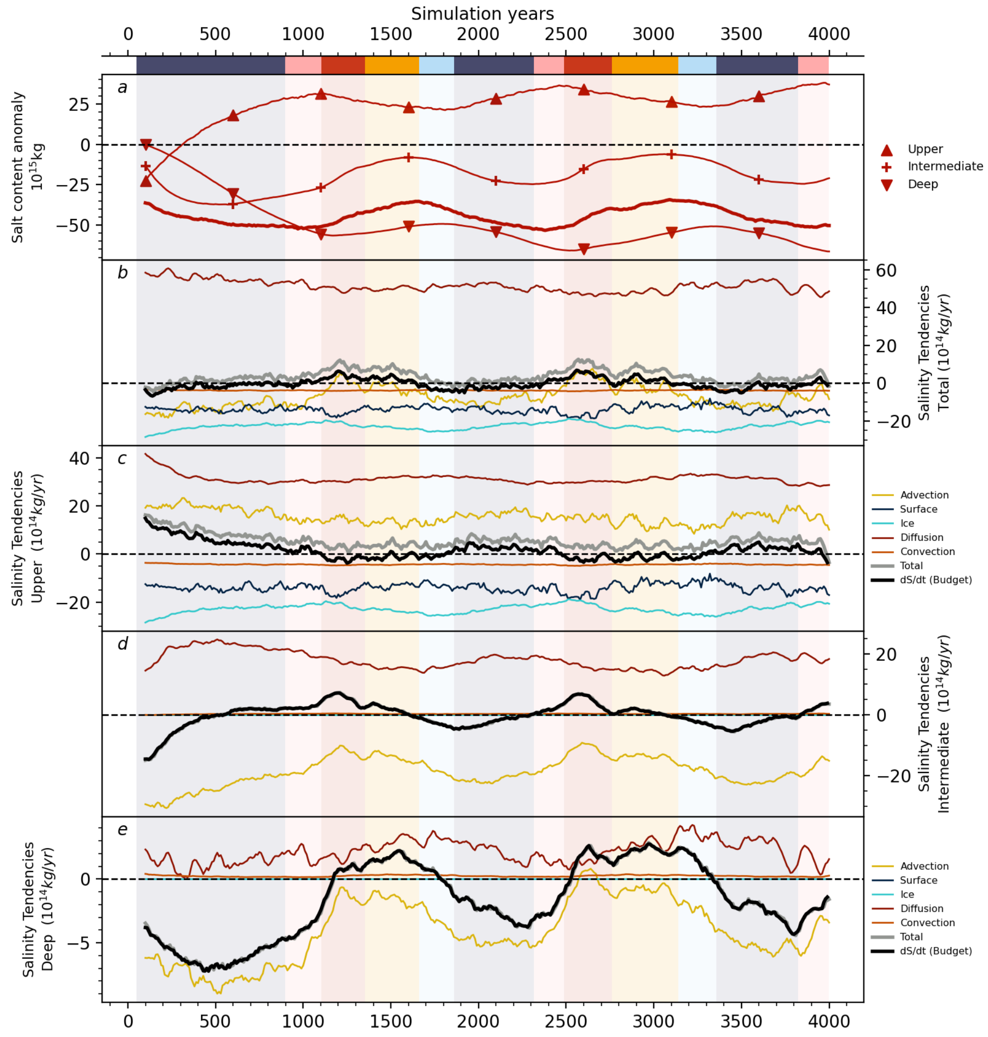

Fig. S10 Salinity tendencies in the Pacific. (a) Salt content anomaly for the upper, intermediate and deep waters in the **Pacific** (see Figure S1). (b-e) 50-year running means of the absolute salinity tendencies in the region for the entire water column (b), the upper waters (c), the intermediate waters (d) and the deep waters (e). The detail of the tendency fields is given in Section S6. The field *total* corresponds to the sum of all tendencies, and the field dS/dt corresponds to the salt content variations calculated in HadCM3.

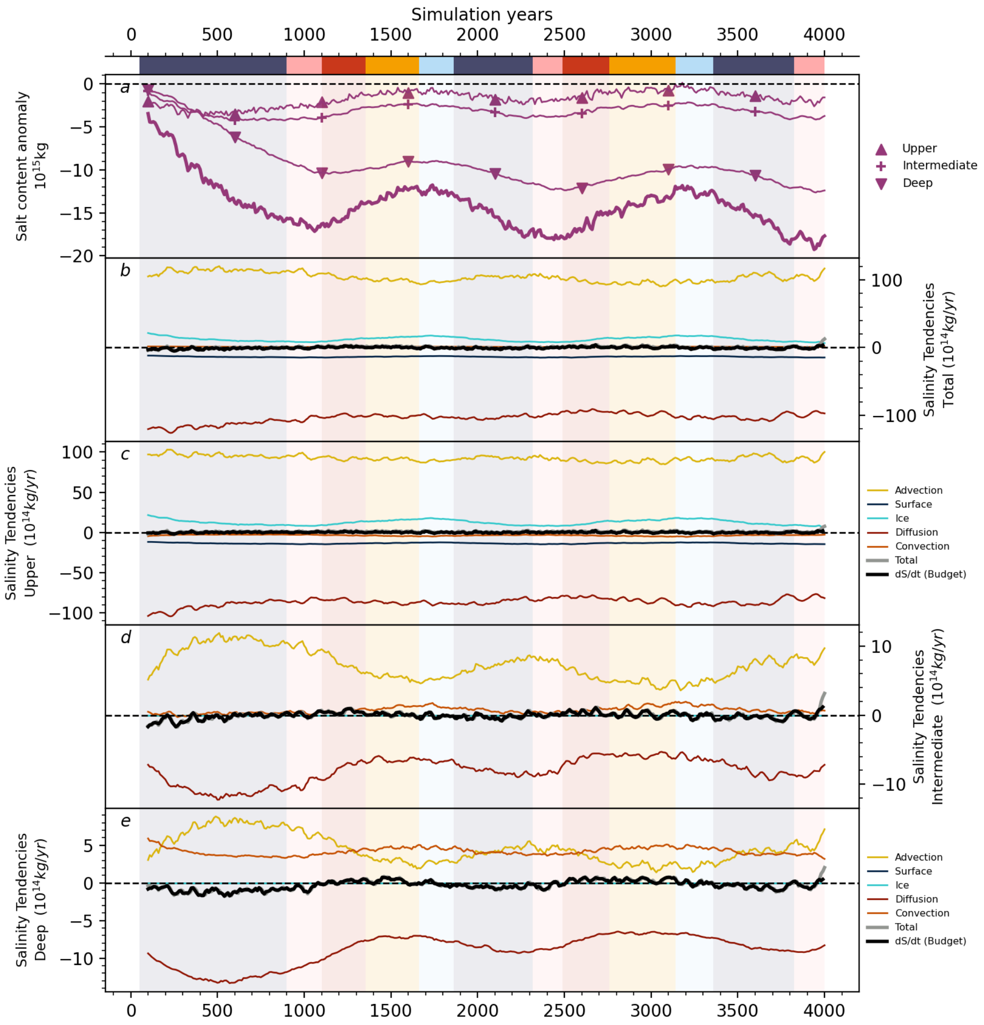

Fig. S11 Salinity tendencies in the Southern Ocean. (a) Salt content anomaly for the upper, intermediate and deep waters in the **Southern Ocean** (see Figure S1). (b-e) 50-year running means of the absolute salinity tendencies in the region for the entire water column (b), the upper waters (c), the intermediate waters (d) and the deep waters (e). The detail of the tendency fields is given in Section S6. The field *total* corresponds to the sum of all tendencies, and the field *dS/dt* corresponds to the salt content variations calculated in HadCM3.

S7 Additional figures for the convection-advection oscillator mechanism

This section contains the additional figures referenced in Section 5 that where not deemed essential to be added to the main text. This includes the vertical temperature, salinity and density profiles in the deep water formation sites during the *warming* phase (Figure S12) and the *cooling* phase (Figure S13), the evolution of the AABW during the *20.7k* simulation (Figure S13), the general concept for millennial-scale variablity mechanism (Figure S15).

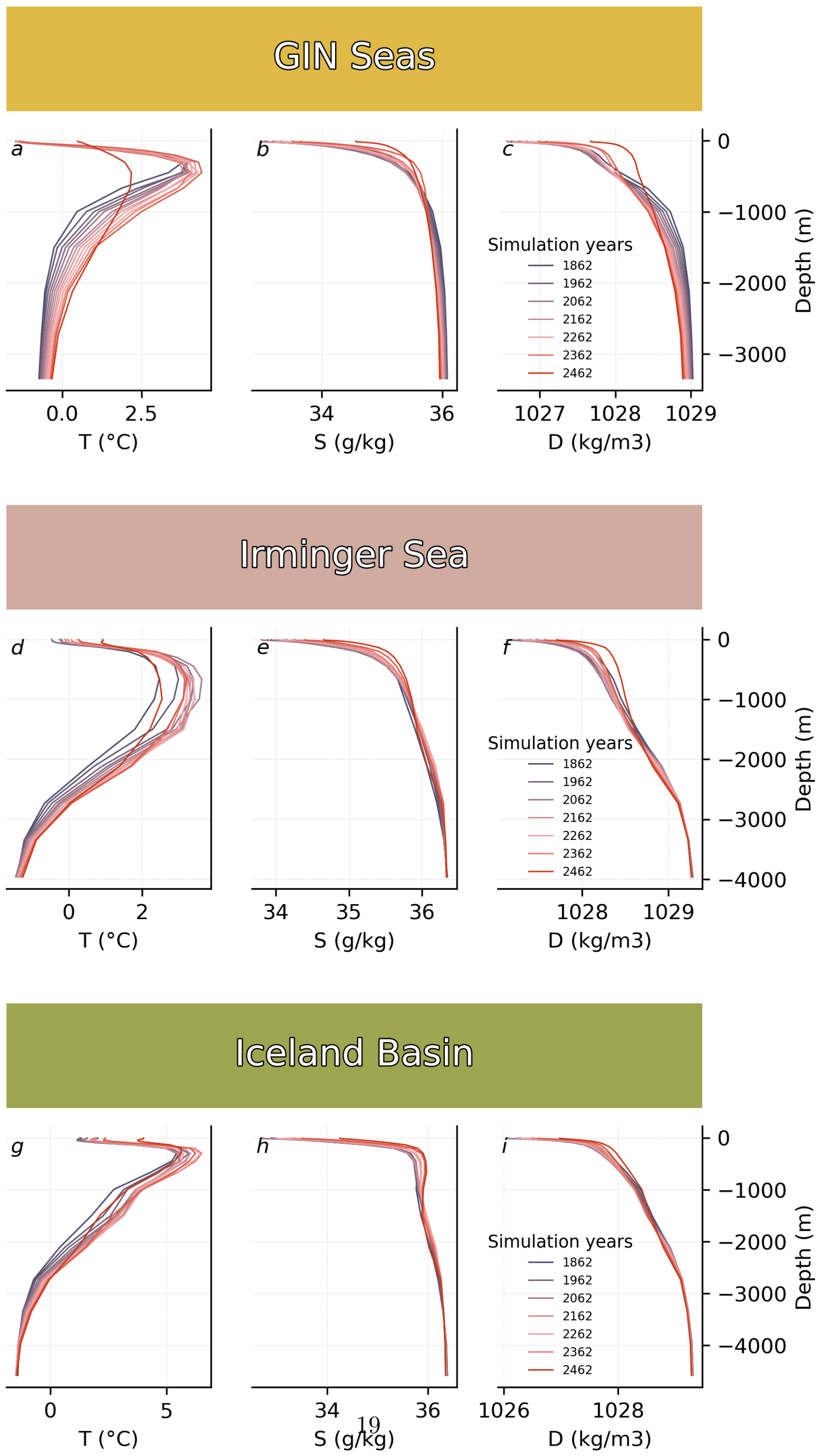

Fig. S12 North Atlantic vertical profiles, warming phase. Vertical profiles of temperature (*a,d,g*), salinity (*b,e,h*) and density (*c,f,i*) in the GIN seas (*a-c*), the Irminger Sea (*d-f*) and the Iceland Basin (*g-i*) every 50 years during the *warming* phase of the *20.7k_tdc* simulation.

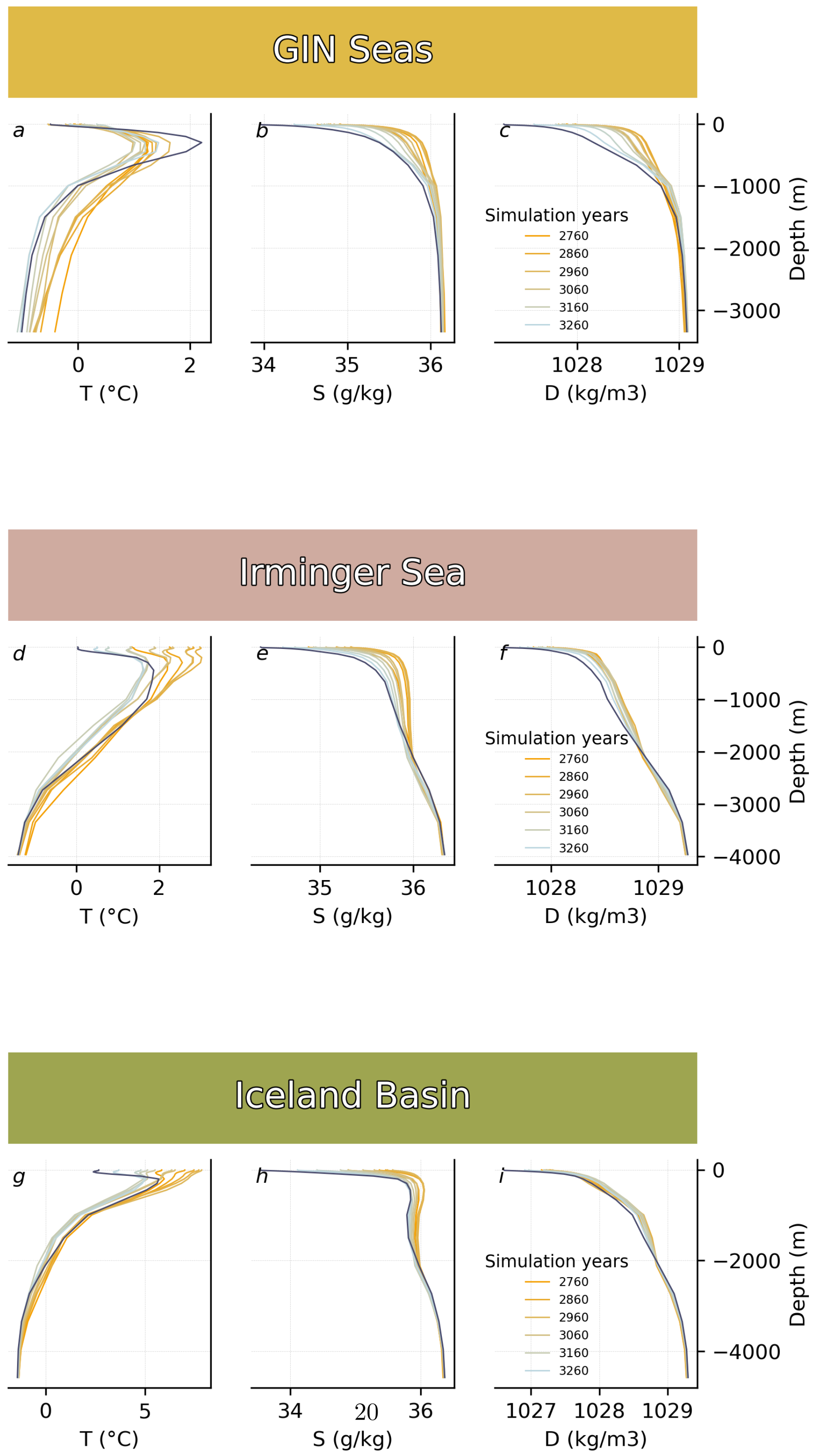

Fig. S13 North Atlantic vertical profiles, cooling phase. Vertical profiles of temperature (*a,d,g*), salinity (*b,e,h*) and density (*c,f,i*) in the GIN seas (*a-c*), the Irminger Sea (*d-f*) and the Iceland Basin (*g-i*) every 50 years during the *cooling* phase of the *20.7k_tdc* simulation.

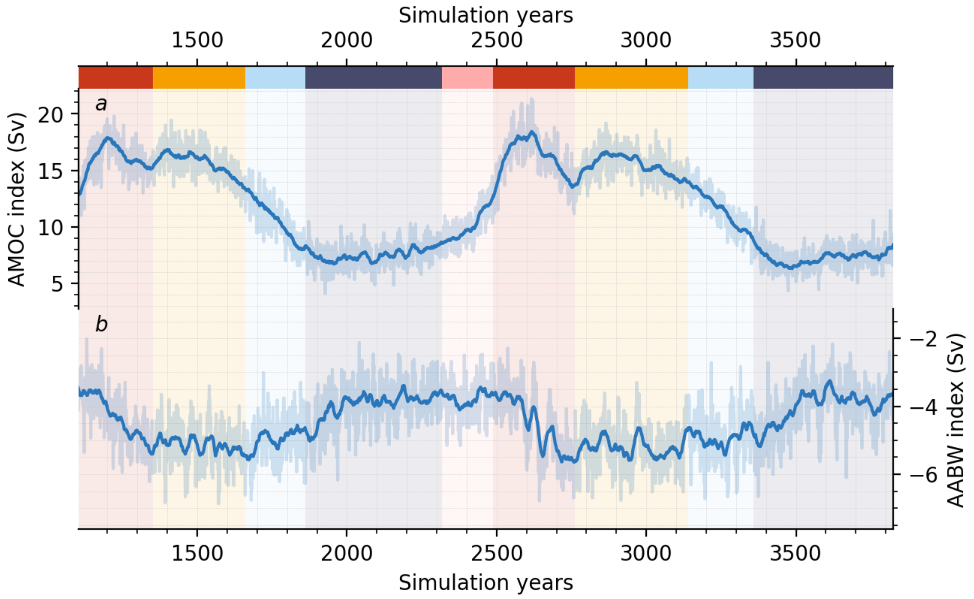

Fig. S14 Antarctica bottom waters index. *a.* AMOC index of the *20.7k_tdc* simulation defined as the maximum overturning circulation at 26.5° N. *b.* AABW index of the *20.7k_tdc* simulation defined as the minimum overturning circulation at 33° S.

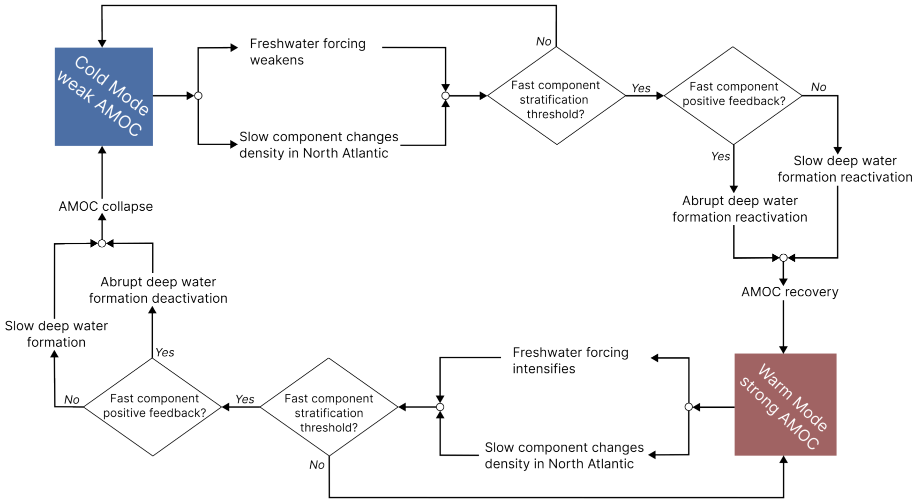

Fig. S15 A general concept for millennial-scale variability.

S8 The influence of the salinity conservation algorithm

In Section 6, we discuss the role of the mean global salinity conservation algorithm, and the drifts that can occur when it is not activated in HadCM3 simulations. Figure S16 provides a comparison of the AMOC index, the salinity mean and salt content budget, the stratification index and the temperature profile of the *20.7k* simulation and the *20.7k* simulation with the global salinity target turned off (*20.7k_no_gst*).

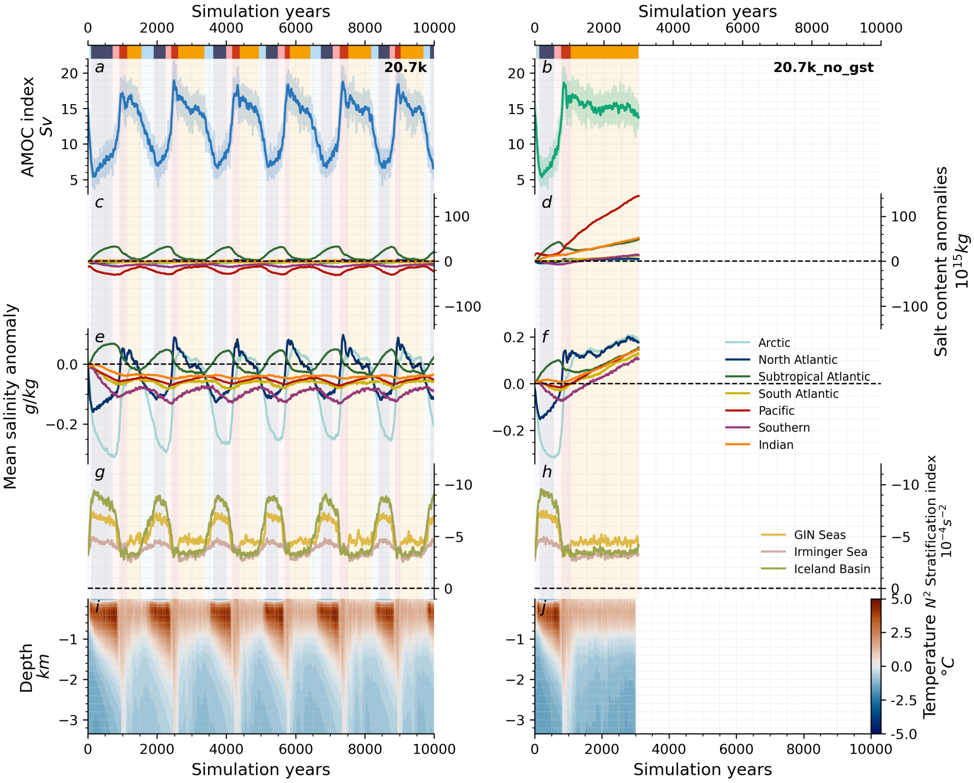

Fig. S16 **The role of the global salinity target**. 30-year running means (solid lines) and annual mean (transparent lines) time series for (*a*-*b*) maximum AMOC strength, (*c*-*d*) salinity budgets in global ocean basins (see Figure 3*a*), (*e*-*f*) mean salinity in global ocean basins, (*g*-*h*) N2 stratification index (Li et al, 2020) in the deep water formation sites (low negative values indicate high stratification) and (*i*-*j*) ocean potential temperature profiles in the North Atlantic for the *20.7k* and the *20.7k_no_gst* simulations, in analogy to Figure 5

References

Armstrong E, Izumi K, Valdes P (2022) Identifying the mechanisms of DO-scale oscillations in a GCM: a salt oscillator triggered by the Laurentide ice sheet. Climate Dynamics https://doi.org/10.1007/s00382-022-06564-y, URL https://doi.org/10.1007/s00382-022-06564-y

Gent PR, McWilliams JC (1990) Isopycnal Mixing in Ocean Circulation Models. Journal of Physical Oceanography 20(1):150–155. https://doi.org/10.1175/1520-0485(1990)020⟨0150:IMIOCM⟩2.0.CO;2, URL https://journals.ametsoc.org/view/journals/phoc/20/1/1520-0485_1990_020_0150_imiocm_2_0_co_2.xml, high - Read

Ivanovic RF, Valdes PJ, Flecker R, et al (2013) The parameterisation of Mediterranean–Atlantic water exchange in the Hadley Centre model HadCM3, and its effect on modelled North Atlantic climate. Ocean Modelling 62:11–16. https://doi.org/10.1016/j.ocemod.2012.11.002, URL https://www.sciencedirect.com/science/article/pii/S146350031200162X

Ivanovic RF, Valdes PJ, Gregoire L, et al (2014) Sensitivity of modern climate to the presence, strength and salinity of Mediterranean-Atlantic exchange in a global general circulation model. Climate Dynamics 42(3):859–877. https://doi.org/10.1007/s00382-013-1680-5, URL https://doi.org/10.1007/s00382-013-1680-5

Li G, Cheng L, Zhu J, et al (2020) Increasing ocean stratification over the past half-century. Nature Climate Change 10(12):1116–1123. https://doi.org/10.1038/s41558-020-00918-2, URL https://www.nature.com/articles/s41558-020-00918-2, number: 12 Publisher: Nature Publishing Group

Romé YM, Ivanovic RF, Gregoire LJ, et al (2022) Millennial-Scale Climate Oscillations Triggered by Deglacial Meltwater Discharge in Last Glacial Maximum Simulations. Paleoceanography and Paleoclimatology 37(10):e2022PA004451. https://doi.org/10.1029/2022PA004451, URL https://onlinelibrary.wiley.com/doi/abs/10.1029/2022PA004451
